# HBV sequence integrated to enhancer acting as oncogenic driver epigenetically promotes hepatocellular carcinoma development

**DOI:** 10.1186/s13046-025-03413-8

**Published:** 2025-05-22

**Authors:** Lu Chen, Wenxuan Li, Wenjing Zai, Xiangyi Zheng, Xianlong Meng, Qunyan Yao, Wei Li, Ying Liang, Mu Ye, Kaicheng Zhou, Mengxing Liu, Zhicong Yang, Zhanrui Mao, Hongyan Wei, Shuai Yang, Guoming Shi, Zhenghong Yuan, Wenqiang Yu

**Affiliations:** 1 Shanghai Public Health Clinical Center & Laboratory of RNA Epigenetics, Institutes of Biomedical Sciences & Department of General Surgery, Huashan Hospital, Cancer Metastasis Institute, Shanghai Medical College, Fudan University, Shanghai, China; 2 Key Laboratory of Medical Molecular Virology (MOE/NHC/CAMS), Research Unit of Cure of Chronic Hepatitis B Virus Infection (CAMS), Shanghai Frontiers Science Center of Pathogenic Microbes and Infection, School of Basic Medical Sciences, Shanghai Medical College, Fudan University, Shanghai, China; 3 Liver Cancer Institute, Key Laboratory of Carcinogenesis and Cancer Invasion, Ministry of Education, Zhongshan Hospital, Fudan University, Shanghai, China; 4 Department of Liver Surgery and Transplantation, Department of Gastroenterology and Hepatology, Zhongshan Hospital, Fudan University, Shanghai, China; 5 Precision Pharmacy and Drug Development Center, Department of Pharmacy, Tangdu Hospital, Fourth Military Medical University, Xi’an, China; 6 Research and Development Department, Shanghai Epicurer Biotechnology Co., Ltd., Shanghai, China

**Keywords:** HBV Sequences Integrated To Enhancer (HBV-SITEs), Nuclear activating miRNA, Enhancer, Hepatocellular carcinoma (HCC), HBV integration

## Abstract

**Background:**

HBV integration is considered as the main contributor to hepatocellular carcinoma (HCC). However, whether HBV integrated sequences determine genotype pathogenicity and how to block their function during HCC progression remains unclear.

**Methods:**

An in vitro HBV-infected PHH model and liver cancer cell lines were established to confirm the pathogenic potential of HBV-SITEs. The roles of HBV-SITE-1 in HCC development were analyzed using cellular phenotypic assays and molecular biology techniques, including the combined analysis of RNA-seq and ChIP-seq. Animal models were also used to evaluate the therapeutic effect of HBV-miR-2 inhibitors.

**Results:**

We identified nine fragments of **HBV S**equences **I**ntegrated **T**o **E**nhancer, termed as “HBV-SITEs”. Particularly, a single nucleotide variation (T > G) was embedded at seed sequence of HBV-miR-2 in the highest integrated HBV-SITE-1 between genotypes B and H. Unexpectedly, B-HBV-SITE-1, not H-HBV-SITE-1, could abnormally activate oncogenic genes including *TERT* and accelerate HCC cell proliferation and migration. Meanwhile, HBV-miR-2 was gradually increased in HBV-infected cells and patient plasma with different HCC stages. Importantly, 227 genes upregulated by HBV, were also activated by HBV-miR-2 through triggering HBV-SITE-1 enhancer. Conversely, enhancer activities were particularly decreased by HBV-miR-2 inhibitors, and further downregulated activated oncogenic genes. Finally, HCC growth was dramatically restrained and HBV-induced transcripts were systematically reduced via injection of HBV-miR-2 inhibitors in animal models.

**Conclusion:**

HBV-SITEs were identified as novel oncogenic elements for HCC, which provides an insightful perspective for the other cancers caused by oncogenic DNA viruses. We demonstrated that the integrated HBV sequence itself acted as oncogenic enhancers and nucleotide variations of HBV genotypes account for particular pathogenic progression, supporting that the viral nucleotide sequences are vital pathogenic substances beyond viral proteins. And modulation of their enhancer activities could be clinically achievable strategy for blocking DNA viruses-related cancer progression in the future.

**Supplementary Information:**

The online version contains supplementary material available at 10.1186/s13046-025-03413-8.

## Background

HBV genotypes B and C are prevalent in China and genotype H is mainly circulated in Central and South America [1, 2]. Notably, HBV genotypes B or C infection appeared 69.9% of HCC in China while genotype H infection was identified with a low incidence of liver disease and HCC in Central America [3]. Surprisingly, there are only 4 ∼ 7.9% sequence differences among these HBV genotypes [4]. Therefore, paying more attention to the minute nucleotide sequences will help us to explore the oncogenicity of different HBV genotypes. HBV infection remains an important public health problem due to its contribution for liver fibrosis, liver cirrhosis and hepatocellular carcinoma (HCC) [5]. Notably, HBV DNA integration into the human genome occurred in 85 ∼ 90% of HBV-related HCC [6], thus it has been clearly demonstrated that HBV DNA integration is the main contributor to HCC. Specially, the integration of HBV DNA as an early step of clonal tumor expansion could induce genomic instability and further activate oncogenic pathways to promote tumorigenesis [7]. Meanwhile, HBV encoding proteins such as HBsAg and HBx act as pathogenic factors [8, 9]. In addition, abundant evidence has demonstrated that virus-derived miRNAs from noncoding viral regions could act as vital elements to modulate viral and host gene expression for diseases development [10–12]. However, little is known whether HBV integrated non-coding sequence itself could act as a regulator for HCC development and determine the HBV genotypes pathogenicity.

It has been revealed that oncogenic viruses including HBV, HPV and EBV are all highly adapted to maintain chronic infections in human and have evolved to persist in their host for years to promote tumorigenesis [13]. During this long-term procedure, viral sequences could integrate into the human genome and further drive the multistep tumorigenesis from uncontrolled proliferation to transformation [13], indicating that silencing pathogenic integrated sequences at appropriate times may stop or reverse viruses-related disease progression. HBV genomic integration accelerated the hepatocellular carcinoma (HCC) development from liver cirrhosis to oncogenesis [14]. While interferon and nucleoside drugs could decrease the viral burden for patients infected with HBV [15], it is difficult to absolutely eliminate the HBV from patients after HBV integrated into human genome [16], and silencing integrated HBV DNA remains a therapeutic challenge. Accordingly, discovering novel therapeutic target is important to combat against HBV integration for blocking HCC progression. Importantly, primary hepatocytes are used as the gold standard for HBV investigation in vitro, but they are not widely used in experimental research due to limited expansion and complexed culture conditions [17, 18]. Of note, we have reported that the 5 C culture conditions can enable primary hepatocytes to be cultured for 28 days to support long-term infection of HBV [19]. Therefore, it provides us the models to explore the molecular procedure of hepatocarcinogenesis caused by long-term infection of HBV and verify the therapeutic target for blocking HCC progression.

Enhancers are cis-regulatory elements bound by transcription factors (TFs) and coactivator complexes and marked with specific post-translational modifications (PTMs) such as H3K4me1 and H3K27ac [20]. Altered enhancers could regulate their spatial and temporal gene expression around their genomic regions during cancer development [21–23], and gains and losses of enhancer activity were associated with the tumor initiation and metastatic transition [24]. We previously demonstrated that the noncoding sequences such as NamiRNAs, as an enhancer trigger, could crosstalk with enhancers in regulating gene transcription for breast cancer development, and virus non-coding HIS sequence also could target host enhancer regions to upregulate genes during COVID-19 progression [25–28]. Accordingly, it is important to investigate the crosstalk between human enhancers and HBV integrated sequences for understanding the HBV oncogenicity. Furthermore, it has been revealed that inhibitors or antagomirs targeting miRNAs could inhibit gene expression by decreasing enhancer activities [25, 29], which provides the applicable approaches that modulating enhancer activity may be a promising strategy for HCC therapy.

Herein, we found that there existed nine fragments of HBV genomic sequences tend to particularly insert into human enhancer regions, and named as “HBV-SITE”. And we picked HBV-SITE-1 embedded with HBV-miR-2 to investigate its tumorigenic ability due to its highest integrated frequency (20.40%). Clearly, the upregulated 1504 genes by HBV-SITE1 from genotype B (B-HBV-SITE1) were enriched in tumor-promoted pathways. Notably, B-HBV-SITE1 acting as an enhancer dramatically stimulated HCC cell proliferation and migration, which could be significantly blocked by reducing its enhancer activities through HBV-miR-2 inhibitor. Instead, HBV-SITE-1 from genotype H (H-HBV-SITE1) couldn’t reinforce cell proliferation and migration in HCC. Subsequently, we established a long-term HBV-infected PHH cell model to partially mimic HCC progression with particular gene expression patterns. Interestingly, HBV-miR-2 transcribed from HBV-SITE-1 was gradually elevated in PHH during HBV infection and progressively increased in patient plasmas from CHB, liver cirrhosis, to HCC. On the contrary, transfection of HBV-miR-2 inhibitor in HBV-infected PHH could downregulate tumor genes expression involved in angiogenesis (*CD34*) through decreasing enhancer activity. Further animal models demonstrated that antagomir treatment of HBV-SITE-1 in vivo significantly restricted HCC growth and inhibited gene expression in HBV-infected mice. Our findings illustrated that HBV-SITEs may serve as the major contributors for hepatocellular carcinoma development via enhancer-mediated gene activation, and small nucleotide drugs targeting HBV-SITEs may provide an effective approach for the treatment and blockade of HCC progression.

## Methods

### Cell lines and plasmids

Human liver cancer cell lines Huh7, HepG2, and human embryonic kidney cells HEK-293T were cultured in DMEM (HyClone) medium supplemented with 10% fetal bovine serum (HyClone) and 1% penicillin/streptomycin (HyClone). Culture conditions are in a 5% CO_2_ incubator at 37°C. HepAD38 and HepG2-NTCP cells were maintained with Dulbecco’s modified eagle medium (DMEM) supplemented with 10% fetal bovine serum (FBS), penicillin-streptomycin, and tetracycline. Primary human hepatocytes (PHHs) were purchased from Bioreclamation IVT, and cultured as previously described. In brief, cells were thawed and resuspended with a plating medium, and then seeded into collagen I-precoated plates. The PHHs were then cultured with 5 C medium (William’ medium E containing B27, GlutaMAX, penicillin-streptomycin, supplemented with Forskolin (20 µM), SB431542 (10 µM), IWP2 (0.5 µM), DAPT (5 µM) and LDN193189 (0.1 µM)). The supernatants were changed or collected every 3 days.

We obtained a fragment of about 250 bp HBV integrated sequence by annealing and extension, and inserted this sequence into the lentiviral vector pCDH-CMV-MCS-EF1-copGFP at EcoRI (5') and BamHI (3') sites through ClonExpress II One Step Cloning Kit (Vazyme, Cat# C112) according to the manufacturer’s manual. The constructed vectors were used to stable transfection.

### Chemical reagents and antibodies

The inhibitor and control NC of HBV-miR-2 used in the experiments were purchased from RiboBio (Guangzhou, China). Transfection of miRNA inhibitors was performed using the Hieff Trans™ Liposomal Transfection Reagent (Yeasen, China) according to the manufacturer’s instructions.

### Lentiviral packaging and cell screening

We co-transfected pCDH-HBV-SITEs, pSPAX2 (RRID: Addgene_12260), and pMD2.G (RRID: Addgene_12259) plasmids into HEK293T cells at a ratio of 4:3:1.2 and changed serum-containing medium for 6–8 h according to the instructions. The supernatant was obtained by filtration with a 0.45 μm filter for 48–72 h after transfection. Cells were then infected with different lentiviruses to obtain cell lines stably transfected with HBV-SITEs by flow screening and puromycin selection.

### RNA extraction and quantitative RT-PCR (RT-qPCR)

Total RNA was isolated from freshly harvested cells using TRIzol reagent (Invitrogen, 15596018). RNA purity and concentration were assessed by NanoDrop ND-2000 (Thermo Scientific). RNA was reverse-transcribed to cDNA using the PrimeScriptTM RT reagent Kit (Takara, Cat# RR047A). Quantitative PCR was performed using SYBR Green Pre-Mix (TIANGEN, Cat# FP205) on the LightCycler 480 II Real-Time PCR System instrument (Roche). GAPDH and U6 were used as internal reference genes and the relative expression of HBV-SITEs and genes was calculated according to 2^−ΔΔct^ method.

### ChIP-seq and ChIP-qPCR

ChIP experiments were performed as described in our previous research paper [25]. Briefly, the stable transfected cells were cross-linked by formaldehyde, and then sonicated. The cell extract was incubated with antibody against H3K27ac and Protein A Dynabeads (Invitrogen) at 4℃ overnight. DNA was extracted with the Qiagen DNA purification kit (QIAGEN, Cat# 28106). The obtained DNA was subjected to ChIP-qPCR experiments or to obtain a ChIP DNA library for ChIP-seq.

A total of 30 ng prepared DNA templates were used to build libraries for ChIP-seq. ChIP-Seq data analysis sequencing reads were aligned to human genome assembly hg38 using Bowtie2 (version 2.2.5). Then duplicate reads were removed with Samtools. HOMER (http://homer.ucsd.edu/homer/ngs/peaks.html) or MACS2(2.2.7.1) was used to find the H3K27ac peaks with the default setting, compared with the control group. The Pygenometracks (3.8) and deeptools (3.5.1) were used for visualization.

### Dual luciferase reporter assays

The potential enhancer regions were amplified using PCR from the genomic DNA of HEK-293T cells and inserted into the pGL3-promoter vector. The constructed plasmid and Renilla luciferase reporter vector pRL-SV40 were co-transfected into HEK293T cells for enhancer activity assays. Cells were lysed 48 h after transfection and analyzed using the Dual-Luciferase Reporter Assay System (Yeasen, China). The relative activity of enhancer regions was defined by the ratio of firefly/Renilla luciferase activities.

### CCK8, EdU, colony formation and transwell assay

The cells overexpressed HBV-SITEs and the control group were plated in 96-well plates with 3000 cells, and measured OD 450 nm at 24 h, 48 h, 72 h, and 96 h after plating. Each well was repeated three times and obtain the average value to assess the growth trend of the cells. The transfected Huh cells were seeded at 1000 cells/well in 6-well plates and cultured for 2 weeks to form colonies. Cell colonies were stained with 0.25% crystal violet to be imaged and counted.

The EdU (5-ethynyl-2′-deoxyuridine) proliferation assay was performed in 6-well plates. Cells were washed with PBS, and then incubated in serum-free DMEM containing 10 µmol/L EdU (Beyotime, China) for 2 h. Cells were fixed, and then underwent staining according to the manufacturer’s instructions. The cells were imaged using fluorescence microscopy, and the number of proliferating cells was averaged to calculate.

The different group cells were plated in the upper chamber of a 24-well transwell at 40,000 cells per well, and cultured in the serum-free medium. In the lower chamber of the small well, 20% FBS-DMEM was added as an attractant, and the cells were incubated for 48 h. Fixed the well with 100% methanol for 15 min, and then stained with 0.1% crystal violet solution. Photographs were taken under the microscope and cells that migrated to the lower surface were counted.

### Tissue and plasma samples

The collected liver cancer tissue samples and plasmas were all from Zhongshan Hospital Affiliated with Fudan University. Studies involving human tumor tissue were conducted in accordance with the protocol approved by the Ethics Committee of Fudan University, and written informed consent was obtained from all patients after institutional review and approval. The obtained tissue samples were ground in liquid nitrogen as soon as possible, and tissue RNA was extracted by TRIZOL (Invitrogen, 15596018). The patient blood samples were centrifuged to obtain plasma, and plasma RNA was extracted using TRIpure LS Reagent (Bioteke Corporation RP1102) in line with the manufacturer’s instructions.

### HBV infection

HBV virions were collected and concentrated 100-fold from the culture medium of HepAD38 cells. In brief, after the removal of tetracycline, HepAD38 cells were maintained in DMEM consisting of 3% FBS and 2% DMSO, the supernatants were collected every 3 ~ 4 days, pooled, and concentrated by centrifugation after 7 ~ 8% polyethylene glycol (PEG) 8000 (Signa-Aldrich) prediction. The concentrated virus stocks were tittered by qPCR using specific primers, aliquoted, and stored at -80 ℃.

HepG2-NTCP were seeded and infected with HBV at 200 genome equivalents per cell in the presence of 2.5% DMSO and 4% PEG8000 for 16 h. The inoculum was then removed, and the cells were washed with PBS 3 ~ 6 times and maintained in the medium containing 2.5% DMSO. The supernatants and cells were collected at indicated time points.

The PHHs cells were infected with HBV at 200 genome equivalents per cell in null mediums (William’s medium E containing B27, GlutaMAX, penicillin-streptomycin) supplemented with 4% PEG8000. The medium was removed 16 h later, the cells were rinsed with PBS 3 ~ 6 times and then cultured with 5 C medium.

### In vivo nude mouse model

Twenty-four BALB/c nude mice (4 ~ 6 weeks old) were divided into 3 groups. Transfected control vector, overexpressed HBV-miR-2 and the antagomir pre-treatment group on the huh7-HBV-miR-2 cell lines were mixed in serum-free DMEM, and each mouse was injected subcutaneously with 100µL DMEM containing 5 × 10^6^ cells. After tumor formation, mice in the antagomir group underwent intra-tumoral injection of antagomir at a concentration of 10 nm each time, once every three days for a total of three times. Volume measurement is performed using vernier calipers during tumor growth. The tumor volume was calculated using the following formula: volume (mm^3^) = 0.5×width^2^×length. On day 18 after injecting cells, the mice were sacrificed to harvest the tumors for further analysis. All animal experiments were approved by the Fudan Committee on Animal Care and in compliance with ethical guidelines.

### Hydrodynamic injection (HDI) of tail vein mouse model

HDI is an important technique to induce the expression of HBV in mice. HDI is used to inject a large volume of solution containing plasmid DNA into the mouse tail vein over a period of 5 to 8 s [30]. The huge water pressure makes the injected solution retrograde into the liver, leading to a rapid increase in liver volume, the increased permeability of the capillary endothelium and the enlarged pores in cell membranes, therefore allowing the plasmid DNA into the liver cells. This method prevents plasmid DNA from being degraded by DNA enzymes in the bloodstream and improves transfection efficiency [31]. In 2002, HDI was used to inject plasmids containing the HBV genome to construct HBV-infected mouse [32]. pAAV carrying the HBV genome can achieve high and sustained expression in mice liver, making them suitable for establishing HBV infection model [33].

Synthetic miRNA antagomir can be diluted with sterilized PBS, and miRNA antagomir with special chemical modification can overcome obstacles such as cell membrane to enter the target cells. Previous research has reported miRNA antagomir administered by intravenous injection could play a role in the treatment of many diseases [34–38].

In order to make miRNA antagomir enter the liver cells efficiently and reduce the damage of HDI injection times to the liver of mice, we carried out co-injection of plasmid DNA and miRNA antagomir. Ten C57BL/6 mice (male, 6 weeks old) were randomly divided into 3 groups, including pAAV8-control + antagomir NC, pAAV8-HBV1.3 + antagomir NC or antagomir HBV-miRNA-2. AAV8 can be efficiently targeted to the liver. Then mixed 10 µg plasmids and 20 nm antagomir in 2 mL PBS and rapidly injected mice through the tail vein in a time of 3 ~ 5 s. One week later, mouse liver and blood were collected to perform experiments such as qPCR, ELISA, and IHC assays.

### Immunohistochemical (IHC) staining

The obtained mouse tumor tissues were fixed with 4% formaldehyde for 48 h, dehydrated with ethanol solutions of different concentrations, and then embedded in paraffin wax and cut into 4 μm sections. Dewaxing hydration, antigen retrieval and occlusion are performed sequentially. Finally, incubated with the primary antibody overnight at 4 °C. Detailed steps are described in previously published papers [25].

### RNA-seq and gene function annotation

The RNA-seq analysis sequencing reads were aligned to the human reference genome hg38 using Tophat2(2.0.12). Read counts were calculated by Salmon (1.4.0). For chronic hepatitis, liver cirrhosis, and HCC samples, expression data were downloaded from the GEO database (GSE114564). Different expression genes (DEGs) and statistical analysis were performed with DESeq2 (version 3.12) in R (version 4.1.0). Fold change > 1.5, FDR < 0.05. For liver cancer samples, expression data were downloaded from The Cancer Genome Atlas (TCGA, https://portal.gdc.cancer.gov/). Using clinical information to classify them into HBV positive group and HBV negative group. Different expression genes (DEGs) and statistical analysis were performed with DESeq2 (version 3.12) in R (version 4.1.0). Foldchange > 1.5, FDR < 0.05. The heatmaps were created using the pheatmap package (version 1.0.12) and the others were visualized using ggplot2 (version 3.4.2). GO and KEGG pathway analysis of differentially expressed genes were performed using DAVID. Visualizations were performed using ggplot2 or http://bioinformatics.com.cn.

### HBV integration analysis

The RNA-seq sequencing reads were aligned to the merged GRChg38/hg38 genome with no alternative chromosomes and HBV genome (MF967563.1) using STAR (2.7.1a) (hybrid genome (host + virus) where the virus genome is considered as an additional chromosome). Chimeric reads were called using STAR parameters ‘--alignIntronMax 1 --chimSegmentMin 25 --chimJunctionOverhangMin 25’. Minimal overhang for a chimeric junction and minimal length of chimeric segment length parameters were set as 25 for analysis of paired-end RNA-seq dataset. Turn off the splicing alignments by using the option --alignIntronMax 1 as suggested. Extracted viral reads from the generated BAM file by samtools.

We took the HBV reference genome as a bin unit of 250 bp, calculated the occurrence of HBV virus integration events in each bin unit, and calculated its proportion to the total number of integrations. Additionally, we download the enhancer regions from the enhanceratlas 2.0 database (http://enhanceratlas.org/). If there is an intersection between the HBV integrated genome location and the enhancer, it is identified as the HBV sequence integrated into enhancer regions. And the significance of the inserted enhancer was obtained by the hypergeometric distribution test. Visualizations were performed using Circos (version 0.69-6) or RCircos(version 1.2.2).

### Survival analysis

The gene expression (FPKM) and survival data of Liver Cancer (LIHC) from The Cancer Genome Atlas (TCGA) database were downloaded from the UCSC Xena browser (https://xenabrowser.net/). Univariate Cox regression analysis was first used to evaluate the association between survival time and the expression level of each gene. Patients were divided into the low-expression group and the high-expression group using survminer packages for each gene. The Log-rank test was used to evaluate the survival difference between the two groups.

### Statistical analysis

Data are presented as Mean ± SD of experiments in at least three biological replicates. The two-tailed student’s t-test and one-ANOVA test were applied, and it was considered as significant when the P-value < 0.05. The hypergeometric test was used to test whether the HBV targeting enhancer was significant. Statistical analyses of clinical samples were performed by a two-tailed Mann-Whitney test. In the figures, **** means *P* < 0.0001, *** means *P* < 0.001, **means *P* < 0.01, and *means *P* < 0.05. The data were shown with the averages and SD of at least three biological replicates. Statistical analysis was carried out with R and visualizations using GraphPad Prism 7.0 software.

## Results

### HBV-SITEs particularly integrate into host enhancer promoting tumorigenic phenotypes

**Fig. 1:**
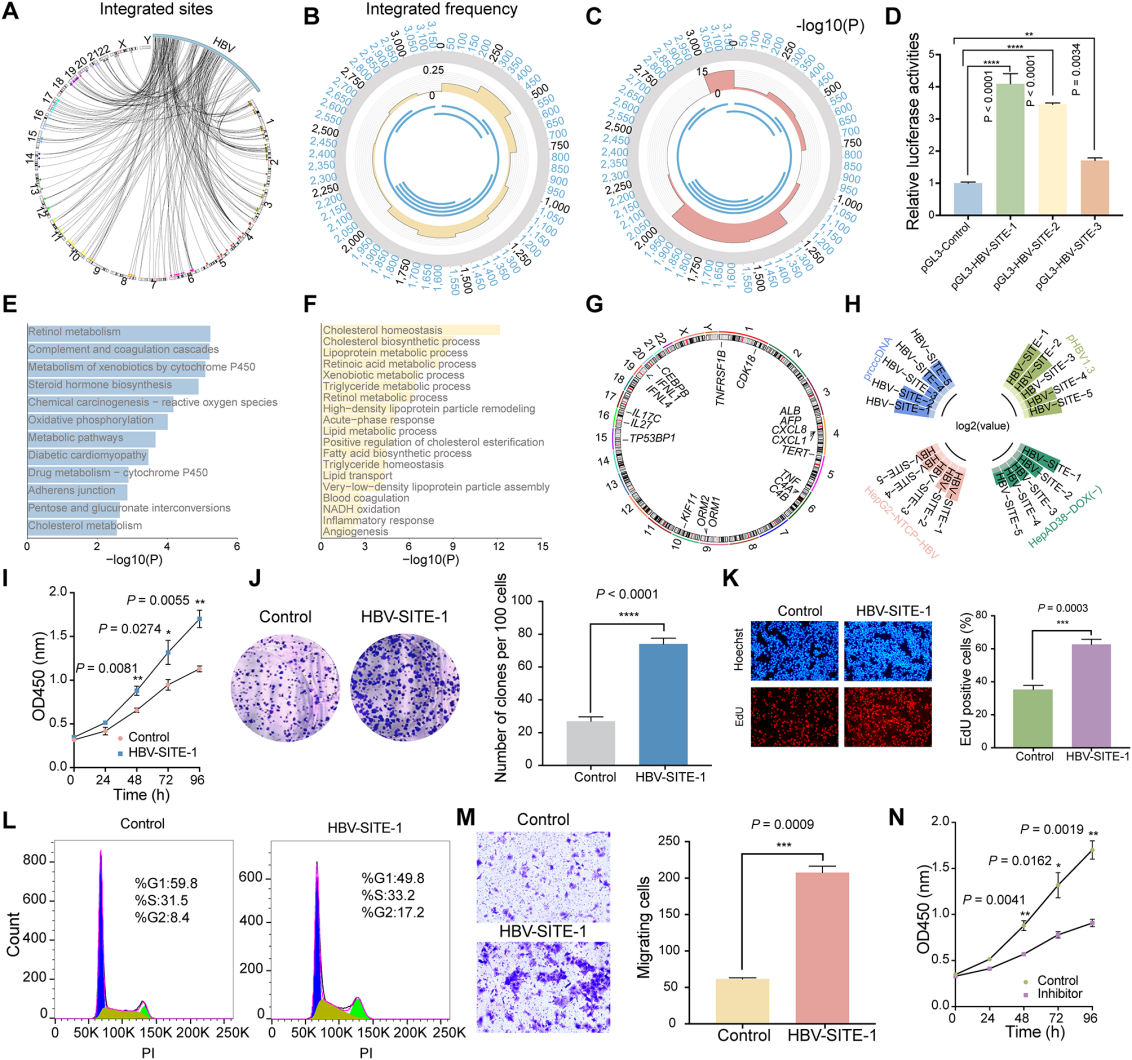
HBV-SITEs promote the HCC cell proliferation and migration. **A** HBV integration events in HBV-infected PHH cells. The junctions between human and HBV sequences were visualized by Circos. The black arrow lines connecting the HBV genome and the human chromosomes at different loci represent the locations of integration. **B** The integration frequency of HBV DNA sequence segmentally. The gray circle from the inside out represents the integration frequency from low to high, and the innermost circle represents the viral protein encoded by HBV. **C** The frequency of the integration sites into human enhancers. The height of pink charts represents the probability of integration into the enhancers. **D** Evaluation of enhancer activities by dual luciferase reporter assays. Transfection of PGL3-HBV-SITEs plasmids in HEK293T cells. **E** Enrichment analysis of the genes surrounding the integration of HBV-SITEs in HBV-infected PHH. **F** Enrichment analysis of upregulated genes in HBV-infected PHH coincided with genes surrounding the integration of HBV-SITEs. **G** Analysis of oncogenic genes near the HBV-SITE-1 with the highest integrated frequency. **H** Transcripts from HBV-SITEs detected by RT-qPCR in four established HBV cell models. **I-J** Assessment of cell proliferation by CCK8 (I) and clone formation assay (J) in Huh7 cells transfected with HBV-SITE-1. **K** Cell proliferation ability evaluated by EdU/Hoechst immunostaining in HepG2 cells after HBV-SITE-1 transfection. **L** Alteration of cell cycle detected by flow cytometry analysis after HBV-SITE-1 transfection. **M** Migration ability detected by transwell assays with transfected HBV-SITE-1. **N** Changed proliferation of liver cancer cells after inhibition of HBV-SITE-1

Accumulated evidence has demonstrated that the host genome stability destroyed by HBV integration is a vital factor to drive oncogenic transformation [6, 39], but we focused on the effect of HBV integrated sequence itself. To analyze the specific fragments of HBV DNA integrated into the human genome, we conducted a model of HBV-infected PHH cells using the 5 C culture assay [19], and the HBV antigen levels increased rapidly and presented a relatively stable high expression after 14 days of infection, indicating that this model worked (Fig. S1A and S1B). Subsequently, we analyzed the human-HBV chimeric reads with our RNA-seq data using previously reported strategy [40, 41] and visualized these data by Circos [42], and there were 353 HBV integration events occurred in HBV-infected PHH (Fig. 1A). As for the inserted sites of host genome, HBV sequences could be inserted into most human chromosomes and seemed relatively random distribution without obvious chromosomal preference. Instead, for the HBV genome itself, these integrated sequences were preferentially in specific regions located at the nucleotides 0 ~ 750 and 1250 ~ 2000 of the HBV genome (Fig. 1B), which was supported by the other reports presented in 426 paired clinical samples [39]. Of note, the integrated sequences at 250 ~ 500 nt and 1500 ~ 1750 nt occupied higher frequency as 20.40% and 15.58% respectively (Fig. 1B), and we speculated that these preferential integrated fragments of HBV may serve as critical pathogenic elements.

Recently, we found that SARS-CoV-2 RNA elements could facilitate COVID-19 progression via enhancer-mediated gene activation [28], proving that virus genomic elements could interact with host enhancers. Alteration of activity or dysfunction of enhancers are known to promote oncogenic gene expression during cancer development [22, 24]. Therefore, we asked whether HBV integrated sequences may act as enhancers or regulate host enhancer activity contributing to hepatocarcinogenesis. We downloaded human enhancers from Enhancer Atlas database and obtained 108 integration events into enhancer regions, and we further discovered that most of HBV integrated sequences were significantly enriched in human enhancer regions (Fig. 1C), implying that HBV specific fragments could orchestrate with enhancer. Meanwhile, we found these HBV fragments could act as enhancers by the result that transfection of these integrated sequences observably increased the luciferase activities (Fig. 1D). Given this special pattern that HBV genome preferentially inserted into host enhancers, we named these specific fragments as **HBV S**equences **I**ntegrated **T**o **E**nhancers (HBV-SITEs) and numbered these nine sequences based on the frequency of integration. For example, HBV-SITE-1 located in 251 ~ 500 nt displayed the highest frequency of 20.40%. We further explored 2729 neighbor genes at the upstream and downstream 500 kb around human enhancers of HBV-SITEs, which were mainly enriched in the metabolic pathway and chemical carcinogenesis (Fig. 1E). Further, we also analyzed upregulated genes in HBV infected PHH cells. Interestingly, among these upregulated genes, 317 genes were overlapped with these 2729 genes surrounding enhancer, which were mainly related to the liver metabolic process, inflammatory response and angiogenesis (Fig. 1F), highlighting that HBV-SITEs may function in tumorigenesis. Interestingly, lots of upregulated genes around HBV-SITE-1 breakpoints in HBV-infected PHH (Fig. 1G) were previously reported relevant to HCC development [43, 44], such as *TERT*, *AFP*, *ALB*, *KIF11*, and *IL8*, supporting that HBV-SITE-1 could exert significant regulatory function on HCC progression. Previous studies have revealed that the expression of miRNA could represent the enhancer activity of its genome locus [25, 29]. In line with this sight, transcripts of HBV-SITE-1 were quantified by RT-qPCR and showed the highest expression levels in four HBV-related cell models including HBV1.3 transfection and HBV-infected HepG2-NTCP cells (Fig. 1H).

To further confirm our previous results in PHH, we established HBV-infected HepG2-NTCP cell model, and found similar integration events of HBV-SITEs with the preference for the host enhancer regions. Specifically, HBV-SITE-1 integrated into host enhancer with the highest frequency of 34.78% more than that of PHH cells (Fig. S1C and S1D), and about 25% (81) genes surrounding the HBV integrated sites were cooccurred with that in the HBV-infected PHH cells. These confirmed results made us to focus on HBV-SITE-1 for its pathogenic effects during HCC progression. Then, we constructed stable cell lines by transfecting HBV-SITE-1 into HepG2 and Huh7 cells to explore its particular pathogenicity. Subsequently, CCK8, clone formation, and Edu staining assays respectively demonstrated that HBV-SITE-1 significantly promoted cell proliferation in HepG2 and Huh7 cells (Fig. 1I, J and K). Meanwhile, HBV-STIE-1 obviously increased the cell proportion of G2 phase (Fig. 1L), implying that HBV-SITE-1 accelerates HCC cell growth via expediting cell cycle. Moreover, HBV-SITE-1 dramatically enhanced the migratory cell numbers of Huh7 in transwell assay (Fig. 1M), proving that HBV-SITE-1 could promote HCC cell malignancy. As these malignant behaviors may be induced by abnormal enhancer activity of HBV-SITE-1, we designed the potential inhibitors to repress its enhancer activities, which substantially decreased the expression of CDK8 and LOXL2 (Fig. S1E and S1F). Furthermore, this inhibitor treatment observably reduced cell growth, the proportion of EdU-positive cells and the migratory cell numbers (Fig. 1N and Fig. S1G, S1H). Therefore, loss-of-function of HBV-SITE-1 could restrain the tumor-related phenotypes induced by HBV-SITE-1.

Together, these results suggested HBV-SITEs probably acted as enhancers to facilitate the oncogenic transformation of HCC through raising the proliferative and migratory abilities of hepatocytes.

### HBV-SITE-1 upregulated oncogenic genes by interacting with host enhancers

**Fig. 2:**
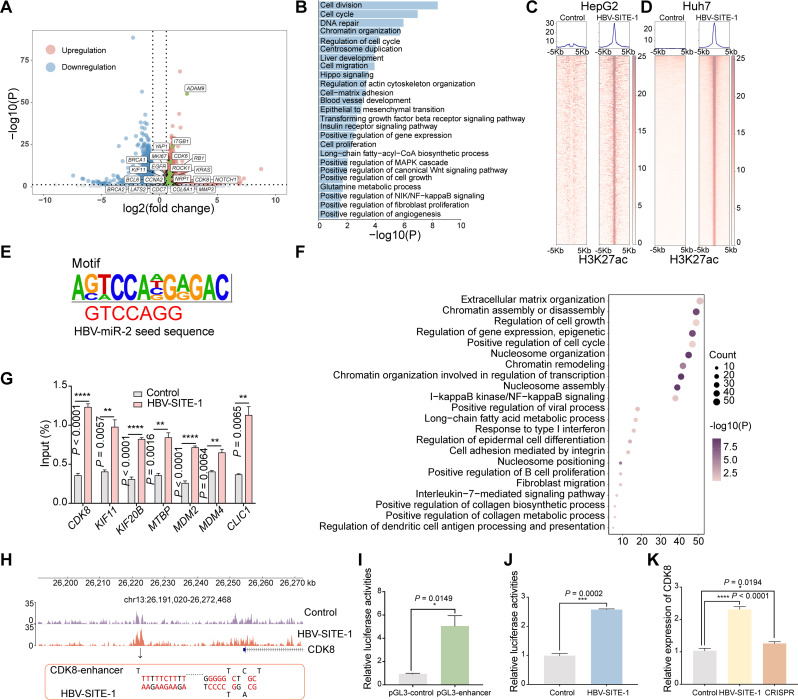
HBV-SITE-1 activates oncogenic gene expression interacting with enhancers. **A** Differential expressed genes in HepG2 caused by HBV-SITE-1. The regulated genes marked in boxes are known oncogenic genes for HCC development. **B** Gene ontology (GO) enrichment analysis of upregulated genes by HBV-SITE-1. **C** Peak enrichment of H3K27ac modification in the HepG2 cells transfected HBV-SITE-1 by HOMER peak calling analysis. Each row represents one peak centered at the midpoint between two 5 kb flanking sequences. **D** Peak enrichment of H3K27ac modification in the Huh7 cells transfected HBV-SITE-1. Each row represents one peak centered at the midpoint between two 5 kb flanking sequences. **E** A motif similar to the sequence of HBV-miR-2 seed sequence is identified in enriched H3K27ac regions induced by HBV-SITE-1. The sequence in red font is the seed sequence of HBV-miR-2. **F** GO enrichment analysis of the genes within upstream and downstream 500 kb centered with the significant enrichment H3K27ac peaks. **G** Enrichments of H3K27ac in the corresponding enhancers with upregulated genes by ChIP-qPCR. **H** H3K27ac enrichment in the corresponding enhancer of *CDK8* targeted by HBV-SITE-1. **I** Activity of the potential enhancers of *CDK8* assessed by Dual-Luciferase Reporter Assay in HEK293T cells after pGL3-*CDK8*-enhancer transfection. **J** *CDK8* specific enhancer activity was increased when transfected HBV-SITE-1 detected by Dual-Luciferase Reporter Assay in HEK293T cells. **K** *CDK8* enhancer mediated genes activation confirmed by CRISPR technology. *CDK8* was decreased after knocking out corresponding enhancer in HBV-SITE-1 transfected HepG2 cells

Based on the significant effect of HBV-SITE-1 on cell behaviors, we asked whether HBV-SITE-1 could affect the transcriptome of liver cancer cells. Accordingly, RNA-sequencing (RNA-seq) revealed that HBV-SITE-1 resulted in the upregulation of total 1504 genes (fold change > 1.5, *P* < 0.05) in HepG2 cells (Fig. 2A), which were mainly enriched in the terms with tumorigenesis including cell division, cell proliferation and migration, and angiogenesis (Fig. 2B and Fig. S2A). In Huh7 cells, HBV-SITE-1 also promoted genes related to cell proliferation and migration, angiogenesis, and further enriched in inflammatory response (Fig. S2B). These similar results in the two cell lines proved that HBV-SITE-1 largely effect in HCC development. It is well-known that inflammation response to HBV infection is an initial inducer for HCC progression during the chronic stage of hepatitis B [45]. In our cell models, a series of chemokines were upregulated more than four folds, such as *CXCL3*, *CXCL8*, *CXCL1*, and *CXCL5* (Fig. S2C), which were connected with poor overall survival rate [46]. Additionally, fibrosis is a repair process to chronic liver injury during the development of hepatic cirrhosis [47]. The upregulated genes including *VCAN* and *ITGAV* by HBV-SITE-1 are both novel biomarkers of hepatitis B virus-related liver fibrosis [48]. Importantly, HBV-SITE-1 also increased the expression of lots of oncogenic genes such as *CDK8*, *LOXL2*, and *HIF1A*, which have been verified to affect HCC patient prognosis [49, 50]. Hence, HBV-SITE-1 was an effective player involved in the processes of HCC development.

Previous studies have revealed that NamiRNAs can activate gene expression by targeting genomic enhancers [25–27, 29]. Meanwhile, exogenous HIS as RNA elements from SARS-CoV-2 could interact with host enhancers to activate genes related to COVID-19 progression [28, 51, 52]. In our models, there were more than 1500 gene upregulations, which may be activated by the exogenous enhancer of HBV-SITE-1 itself, and we are curious whether the transcripts from HBV-SITE-1 could interact with host enhancers to regulate oncogenic genes in the HCC development. Accordingly, we carried out ChIP-seq using the antibody against enhancer marker (H3K27ac) in HBV-SITE-1 cell lines. Clearly, HBV-SITE-1 induced significant 390 peaks enrichment of H3K27ac in HepG2 cells (Fig. 2C), and more than 1500 peaks of H3K27ac in Huh7 cells (Fig. 2D), implying that HBV-SITE-1 could interact with host enhancers and further alter the global enhancer statuses. To figure out the characteristics of these host enhancers activated by HBV-SITE-1, we used motif calling to analyze the 390 enhancer regions in HepG2 and found that this motif sequence embedded the seed sequences (CAGGTCC) of an HBV encoded miRNA-2 (HBV-miR-2) (Fig. 2E), hinting that HBV-miR-2 transcribed from HBV-SITE-1 may act as the mediator for the interaction between HBV-SITE-1 and host enhancers.

As the most enhancers preferentially activate their neighbor genes, we analyzed the genes surrounding these active enhancers (± 500 kb) by HBV-SITE-1, which were expectedly enriched in the terms including cell cycle, metabolic process, as well as interleukin-7-mediated signaling pathway with more than 1600 literature related to cancer (Fig. 2F). Among them, expression of 221 genes were up-regulated by HBV-SITE-1 in our cell models. For instance, cell cycle genes [53–55] (such as *CDK8* and *CLIC1*) were mostly upregulated about four folds in HepG2 cells along with the higher enrichment of H3K27ac at their enhancer regions (Fig. 2G and Fig. S2D). Meanwhile, in Huh7 cells, more than 85% upregulated genes were surrounding around the H3K27ac peaks induced by HBV-SITE-1 (Fig. S2E), which were preferentially enriched in tumorigenic pathways (Fig. S2F). Specially, the upregulated chemokine-related *CXCL10* [56] and pro-oncogenic transcription factor *STAT3* [57] were accompanied with the active enhancers (Fig. S2G), further supporting that HBV-SITE-1 can activate oncogenic genes via interacting with host enhancers.

As cell cycle gene *CDK8*, usually involved in tumor growth, was one of the most potential targets activated by HBV-SITE-1, we performed ChIP-seq and displayed that the H3K27ac peaks were located at about 30 kb upstream of *CDK8* promoter (Fig. 2H), which was verified as active enhancer by ChIP-qPCR (Fig. 2G). To confirm this sequence enhancer potential, the fragments of this region was cloned into the pGL3 vector and found it could function as enhancer by the increased luciferase activities in HEK293T cells (Fig. 2I). Since 15 complementary base pairs existed between the *CDK8* enhancer regions and HBV-SITE-1 through miRanda prediction software with default parameters, we speculated that transcripts from HBV-SITE-1 could interact with *CDK8* enhancer. Thus, we transfected the vector including HBV-SITE-1 as well as pGL3 vector including the *CDK8* enhancer into HEK293T cells, and resulted in strengthening about 2.5 folds of the luciferase activities (Fig. 2J), implying that HBV-SITE-1 transcripts could specifically activate its corresponding host enhancers. As a small-molecule targeting BRD4, JQ1 could block enhancer activities to inhibit gene expression, and *CDK8* was expectedly reduced more than two folds after JQ1 treatment in HepG2 cells (Fig. S2H), indicating that *CDK8* expression was indeed regulated by the recognized enhancer. To further verify whether the *CDK8* enhancer directly mediates this activity in vivo, we knocked out this enhancer region using CRISPR technology and found that transfected HBV-SITE-1 no longer activated *CDK8* expression in HepG2 cells (Fig. 2K), further supporting that the interaction between HBV-SITE-1 and host enhancers regulates oncogenic activation.

Taken together, these findings demonstrated that HBV-SITEs could interact with host enhancers to promote host oncogenic genes during tumorigenesis.

### HBV-SITE-1 (T > G) of genotype H leads to minor pathogenicity

**Fig. 3:**
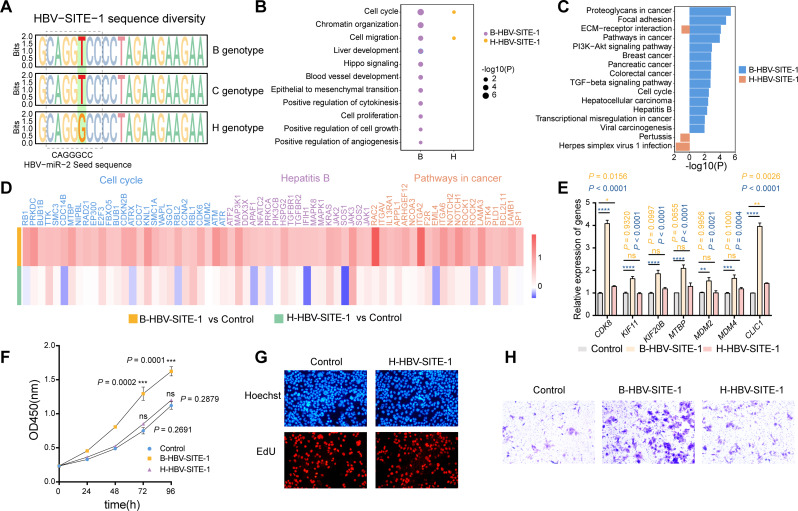
Nucleotide variation of HBV-SITE-1 (T > G) leads to the minor pathogenicity of HBV genotype H. **A** Conservation analysis of the seed sequence of HBV-miR-2 embedded in HBV-SITE-1 among HBV genotypes B, C, and H. The dotted box marks the seed sequence of HBV-miR-2 and the single nucleotide variation (T > G) was marked in green box. **B-C** GO (B) and KEGG (C) analysis of the genes upregulated by HBV-SITE-1 in genotype H compared with genotype B. **D** Among the 113 genes, 66 oncogenic genes displayed were not upregulated by H-HBV-SITE-1 compared to B-HBV-SITE-1. **E** Expression levels of genes induced by H-HBV-SITE-1 detected by RT-qPCR compared to the B-HBV-SITE-1. **F-H** Proliferation and migration ability of HepG2 cells evaluated by CDK8 (F), EdU (G), and transwell (H) assays in H-HBV-SITE-1 compared with B-HBV-SITE-1

As is shown above, HBV-SITE-1 and its transcripts could interact with host enhancers, where embedded a motif similar to HBV-miR-2 seed sequence, and it is noticed that the single nucleotide variation could affect their corresponding enhancer activities and accelerate disease progression [58, 59]. Moreover, it is reported that the prevalent HBV genotype H in the United States is much less pathogenic and carcinogenic than the prevalent B and C in China [3, 60, 61]. Therefore, we performed genomic conservation analysis among HBV genotypes B, C, and H. Compared with the genome of genotype B, there were 284 and 470 nucleotide differences in genotypes C and H, respectively. Surprisingly, in HBV-SITE-1 regions, genotypes B and C had the identical genomic sequences of HBV-miR-2 whereas genotype H altered from T to G coincidently located at the 5th base of its seed sequence (Fig. 3A). Considering that the seed sequences are critical for miRNA regulation, we proposed an adventurous hypothesis that this nucleotide variation (T > G) of HBV-SITE-1 in H genotype (H-HBV-SITE-1) may alter enhancer activities and further account for the pathogenic diversity compared with genotypes B and C.

To prove this hypothesis, we established a stable HepG2 cell line transfected with H-HBV-SITE-1 and performed RNA-seq to compare the differentially expressed genes induced by H-HBV-SITE-1 and B-HBV-SITE-1. Clearly, GO and KEGG analysis revealed that the upregulated genes enriched terms activated by H-HBV-SITE-1 were lost or less relevant to tumor development (Fig. 3B and C). Especially, H-HBV-SITE-1 could not activate 113 genes, including *KRAS*, *ATF2*, *ITGA2*,* EGFR*,* BUB1B*, and *CDC7*, which were enriched in terms of tumorigenesis by B-HBV-SITE-1 (Fig. 3D and Fig. S3), and most of them accounted for poorer survival for HCC patients [62]. Furthermore, RT-qPCR confirmed that these oncogenic genes (such as *CDK8*, *KIF11*, *KIF20*) were not activated by H-HBV-SITE-1 in comparison with B-HBV-SITE-1 (Fig. 3E). Besides, CCK8, EdU staining and transwell assays found that H-HBV-SITE-1 couldn’t significantly influence the cell proliferation and migration caused by B-HBV-SITE-1 (Fig. 3F-H), indicating that the nucleotide variation (T > G) of HBV-SITE-1 largely determines the pathogenic diversity among HBV genotypes B, C, and H. Together, these results further demonstrated that HBV-SITE-1 is a critical pathogenic element for HCC development and its variation may reduce its carcinogenicity of genotype H.

### HBV-miR-2 accelerated HCC progression in 28-day HBV-infected PHH models

**Fig. 4:**
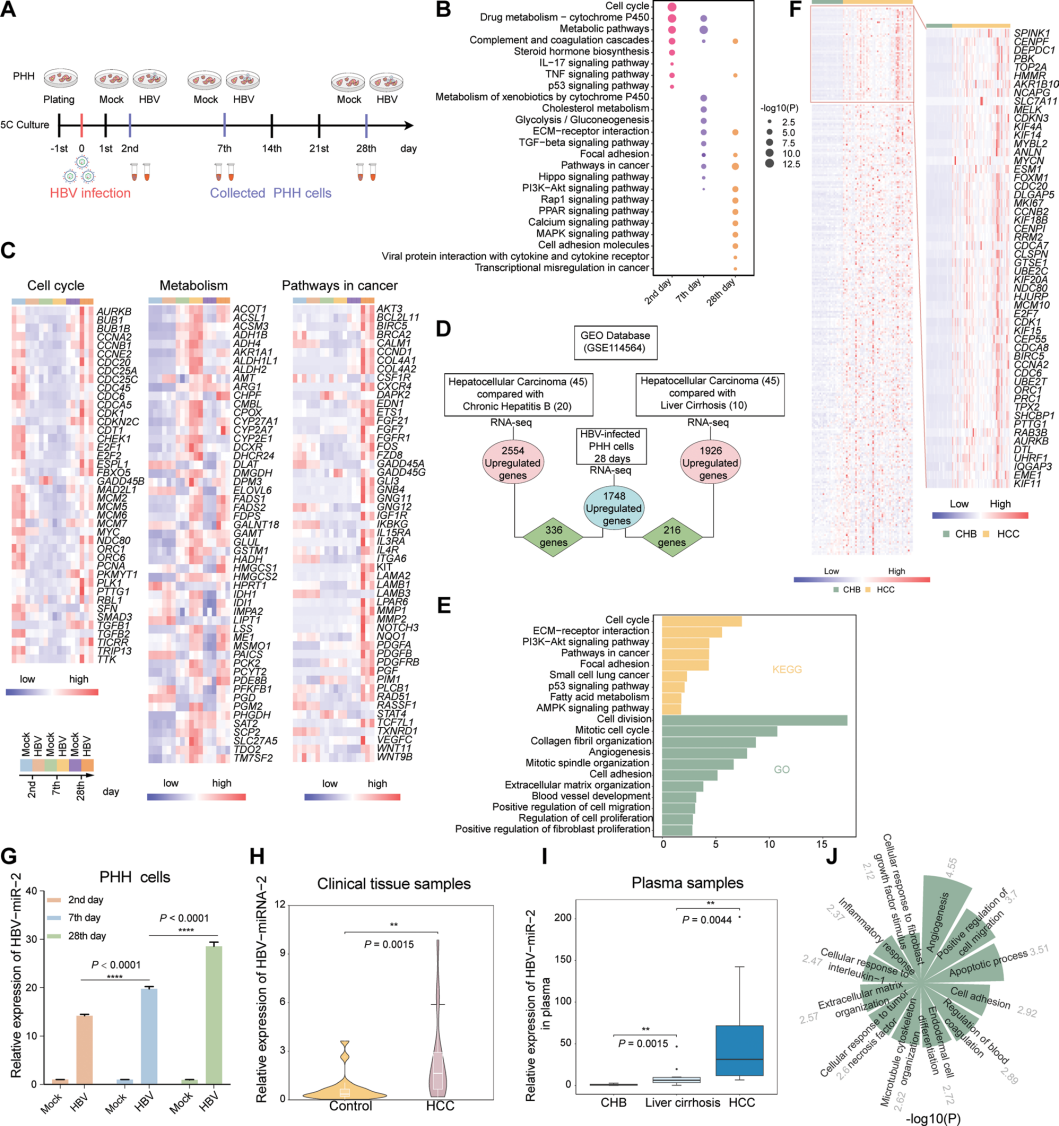
Long-term HBV infection induces particular oncogenic patterns during HCC progression. **A** Schematic of the PHH cells infected with HBV. Cells were obtained after HBV infection for 2 days, 7 days, and 28 days. **B** KEGG Pathway enrichment analysis in HBV infection PHH 2 days, 7 days, and 28 days. **C** Heatmaps of cell cycle, cell metabolism, and cancer pathway for HBV infection 2 days, 7 days, and 28 days. **D** Workflow on the selection of both upregulated genes in clinical samples from the GEO database and HBV-infected PHH cells. In the GEO database, 2554 genes are upregulated in HCC (*n* = 45) compared with CHB (*n* = 20) and 1926 genes were upregulated in HCC compared with liver cirrhosis (*n* = 10). 1748 upregulated genes selected from HBV-infected PHH cells for 28 days. 336 genes are both upregulated between 1748 and 2554 genes, while 216 genes are both upregulated between 1748 and 1926 genes. **E** GO and KEGG analysis on the both upregulated 336 genes selected from HCC clinical databases and HBV-infected PHH cells. **F** Heatmap of 336 upregulated genes were displayed on the left and the top 54 genes (threshold: > 7 folds) presented on the right. **G**, Expression of HBV-miR-2 along with HBV infection detected by RT-qPCR. **H** HBV-miR-2 highly expressed in the tumor tissue compared with the adjacent normal tissues (*n* = 20). **I** HBV-miR-2 detected in higher level in the patient plasma of HCC (*n* = 13) compared with in chronic hepatitis B (*n* = 12) and HBV-related liver cirrhosis (*n* = 11). **J** Gene ontology (GO) analysis of the up-regulated genes in PHH with HBV-miR-2 transfection

It is well-known that HCC progressed sequentially from chronic hepatitis and liver cirrhosis to final HCC formation over the infection period. Primary human hepatocytes are the gold standard for HBV research in vitro [17, 18]. Thus, we asked whether long-term HBV infection in PHH could mimic HCC progression. We infected the PHH with HBV particles obtained from the supernatant of HepAD38, and collected PHH cells at the 2nd, 7th, and 28th days of HBV infection (Fig. 4A). Differentially expressed genes (DEGs) were analyzed in PHH after HBV infection. Clearly, there were three particular gene expression patterns of these DEGs by KEGG enrichment function analysis corresponding to the HBV-infected PHH cells sequentially collected at the 2nd, 7th, and 28th days (Fig. 4B). Then, we further validated this dynamic gene expression by RT-qPCR (Fig. S4A). To further investigate these patterns in detail, we selected genes representing terms as cell cycle, cell metabolism, and cancer pathways to explore their dynamic conversion during HBV infection, respectively (Fig. 4C). In detail, PHH cells infected with HBV in two days mainly affected genes grouped to the cell cycle and metabolism, which may facilitate chronic HBV infection and make hepatocytes prone to subsequent malignant transformations [63, 64]. After 7 days of HBV infection, the DEGs were mostly enriched in alteration of the liver-specific functions such as cholesterol metabolism and glycolysis/ gluconeogenesis, which may change the metabolic microenvironment that could lead to liver fibrosis [65, 66], and abnormal glucose metabolism and high fatty acids appeared in patients with liver cirrhosis [67]. In 28 days of HBV infection, DEGs were significantly enriched in terms with the pathways in cancer, especially in PI3K-AKT and MAPK signaling pathways. Together, as shown, we believed these three particular gene expression patterns were able to mimic and correspond to these three stages of HCC progression.

Furthermore, we confirmed that our 28-day HBV-infected PHH models were able to mimic the progression of HCC by analyzing the differential expressed genes from the GEO database (GSE114564) among the three stages clinical samples including chronic hepatitis B, liver cirrhosis, and HCC. Expectedly, in HCC samples, a total of 2554 genes were up-regulated compared with Chronic hepatitis B while 1926 genes increased compared with liver cirrhosis, respectively (Fig. S4B), indicating that HCC possessed specific transcriptomic signatures. Notably, we found that 336 (coincided with 2554 genes) and 216 (coincided with 1926 genes) upregulated genes were present in PHH after HBV infection 28 days (Fig. 4D). Moreover, these 336 genes were also enriched in the terms related to tumorigenesis (Fig. 4E). Furthermore, these 336 gene expression patterns in 45 HCC samples compared with 20 CHB samples were detailly displayed (Fig. 4F, left) and top 54 genes were also presented (Fig. 4F, right), including well-known prognostic biomarkers of HCC such as *KIF11*, *CCNA2*, *CDK1*, and *CCNB2* [68, 69]. Consistently, we analyzed the clinical samples of HCC induced by HBV from TCGA databases and found that 317 up-regulated genes were coincided with our model at HBV infection 28 days (Fig. S4C), which further demonstrated that long-term HBV infection in PHH cells leads to tumorigenesis. Therefore, our established 28-day HBV-infected PHH models could be used to mimic the progression of HCC.

To further explore the oncogenic effect of HBV-SITE-1 embedded HBV-miR-2 with T to G variation, we evaluated whether HBV-miR-2 expression levels could be a representative marker for HBV-SITE-1 transcripts. Clearly, HBV-miR-2 gradually increased along with the length of HBV infection times, of which showed more 3 folds at 28 days than that in 2 days (Fig. 4G), indicating that HBV-miR-2 expression could reflect the progress of HCC. Accordingly, HBV-miR-2 expression detected by RT-qPCR was significantly higher in the HCC than the adjacent tissues (Fig. 4H). More importantly, HBV-miR-2 expression in plasma progressively increased in HCC patients compared to CHB and liver cirrhosis (Fig. 4I), highlighting that HBV-miR-2 could serve as a potential marker for evaluating HCC progression. Besides, we transfected HBV-miR-2 mimics into primary human hepatocytes and performed RNA-seq. Obviously, the upregulation of 957 genes (such as *MYC*, *CD24*, and *CCL2*) were corrodingly enriched in tumorigenic pathways including angiogenesis, cell migration, inflammatory response, and ECM organization (Fig. 4J and Fig. S4D). Surprisingly, among these 957 genes, 227 genes were also significantly increased in PHH after HBV infection, highlighting that HBV-miR-2 could be a potential target for HBV-infected disease therapy. Notably, HBV-miR-2 activated genes including inflammatory genes like *P2RX7* and *PYCARD* [70, 71], fibrosis-induced genes like *ANXA2* and *MIF* [72, 73] oncogenic genes like *FOS* and *JUN* [74, 75], which were further verified via RT-qPCR (Fig. S4E) and highly expressed in the particular stages of CHB, liver cirrhosis and HCC. Altogether, these results suggested HBV-miR-2 could facilitate the progression of HCC by activating tumor-related genes. In addition to HBV-miR-2 derived from HBV-SITE-1, we found that HBV-SITE-2 transcript mimics in PHH cells could also upregulate 69 genes while HBV-SITE-9 could upregulate 110 genes coincided with HBV-infected PHH models (Fig. S5A and S5B), indicating HBV-SITEs, especially HBV-miR-2 derived from HBV-SITE-1, contributed to HBV pathogenicity.

Together, the 28-day HBV-infected PHH models could mimic the progression of HCC and HBV-miR-2 largely accelerated the HCC progression during HBV infection. Hence, HBV-miR-2 inhibitor may be used for preventing the development of HCC.

### Silencing HBV-SITE-1 enhancer activities by HBV-miR-2 inhibitor

**Fig. 5:**
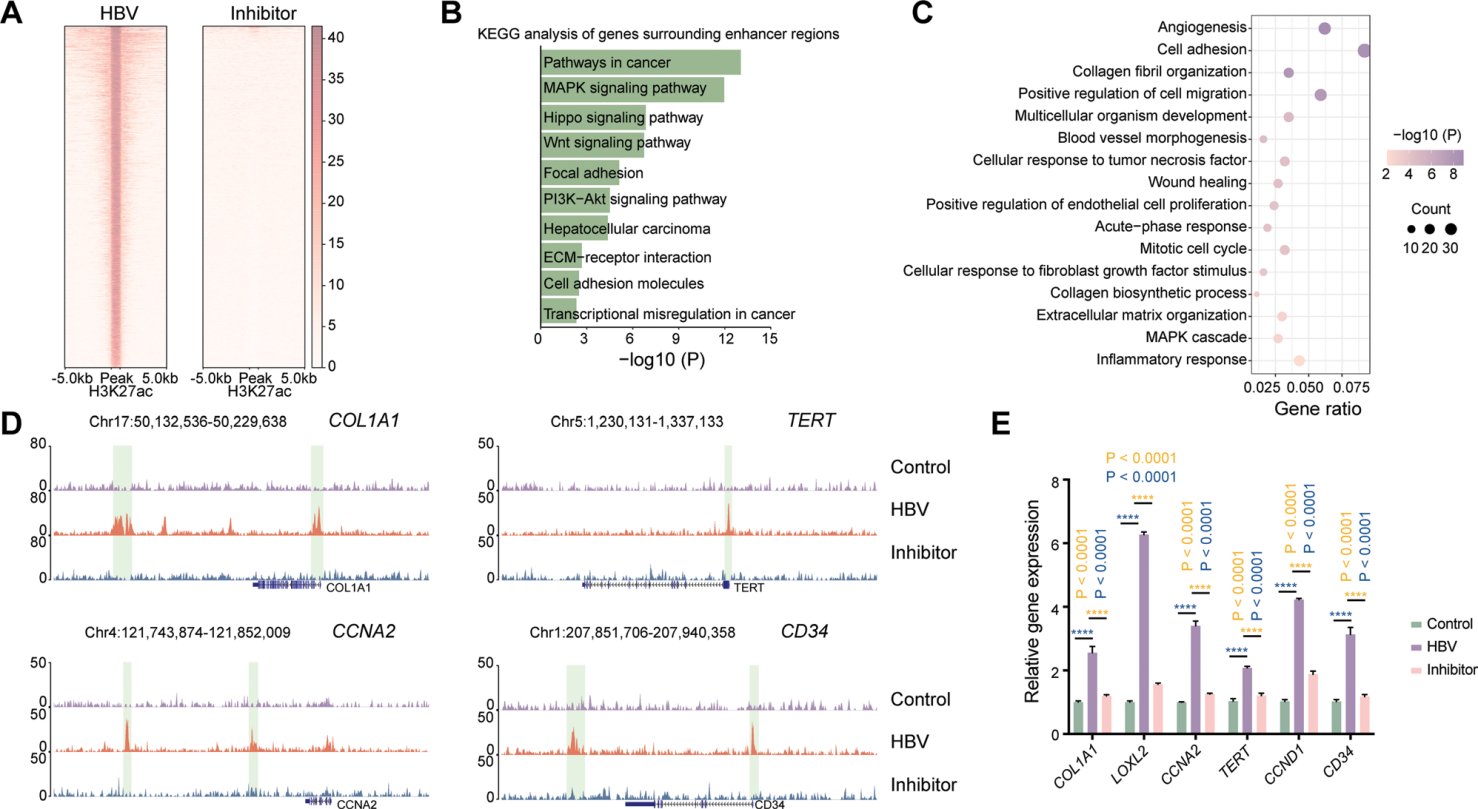
Targeting HBV-SITE-1 downregulates tumorigenic genes through decreasing enhancer activity. **A** Profiling of H3K27ac enrichments in HBV infected HepG2-NTCP cells. Total 5271 H3K27ac peaks in HBV-infected HepG2-NTCP were significantly decreased by HBV-miR-2 inhibitors. Each row represents one peak centered at the midpoint between 5 kb flanking sequences. **B** KEGG analysis of genes surrounding at 3665 enhancers in HBV-infected HepG2-NTCP cells. The 3665 selected enhancers activities were specific targets due to their increases in 5271 H3K27ac peaks induced by HBV infection and simultaneously decreases in 4362 enhancer regions by HBV-miR-2 inhibitor. **C** GO enrichment analysis of the 369 specific genes targeted by HBV-miR-2. The 1305 upregulated genes are caused by HBV infection and the 650 genes are downregulated by HBV-miR-2 inhibitors in HepG2-NTCP cells. The 369 genes are specifically regulated by HBV-miR-2 due to these genes selected from the upregulated 1305 genes and simultaneously downregulated in 650 genes. **D** IGV visualization of H3K27ac peaks in HBV-infected HepG2-NTCP cells. Each peak chart shows the H3K27ac enrichments in control HepG2-NTCP cells (upper), HBV-infected HepG2-NTCP cells (medium), and HBV-infected HepG2-NTCP cells with HBV-miR-2 inhibitor transfection (bottom). The regions on the alteration of enhancer activities are marked in light green boxes. **E** HBV-miR-2 inhibitors specific downregulated genes from HBV infection in HepG2-NTCP cells detected by RT-qPCR

Previous reports have revealed that RNA elements including NamiRNAs and Human Identical Sequences (HIS) can activate gene expression by targeting genomic enhancers, whereas miRNA inhibitors can suppress these gene activations through decreasing the enhancer activities by altering H3K27ac modification [25–29]. We would like to investigate whether artificial miRNA inhibitors could repress the enhancer activities of HBV-SITE-1 to reduce the pathogenicity of HBV during HCC progression. Firstly, we found 1656 up-regulated genes in HBV-infected HepG2-NTCP models. Among these genes, 368 genes were coincided with HBV-infected PHH cells, of which enriched in tumor-related terms (Fig. S6A). Meanwhile, HBV infection could significantly increase H3K27ac modification at 4362 enhancer regions in HepG2-NTCP cells (Fig. S6B), where 79% of the up-regulated genes (1305 among 1656 genes) were located around its 500 kb upstream and downstream (Fig. S6C), indicating that these upregulated genes were enhanced by surrounding active enhancers, and also suggesting that we could use this HBV-infected HepG2-NTCP model for evaluating the silencing of HBV-SITE-1 enhancer by miRNA inhibitors. Therefore, we designed the miRNA inhibitor against HBV-miR-2 and conducted ChIP-seq assays using the H3K27ac antibody in HBV-infected HepG2-NTCP. We infected HepG2-NTCP cells with HBV particles and then transfected HBV-miR-2 inhibitor, and further collected cells to perform ChIP-seq. We found that HBV-miR-2 inhibitor could remarkably decrease the enrichment of H3K27ac at 5271 enhancer regions after HBV infection (Fig. 5A). Then, we compared the locations with the alteration of H3K27ac modification between 5271 and 4362 enhancer regions, and we found there were 3665 enhancer regions coincided among them, of which enhancer activities were potentially silenced by HBV-miR-2 inhibitors. Interestingly, the surrounding genes of these 3665 enhancers were termed with tumorigenesis such as “Pathways in cancer, MAPK signaling pathway, and PI3K-AKT signaling pathway” (Fig. 5B). Expectedly, the 92% down-regulated genes (650 of 707 genes) induced by HBV-miR-2 inhibitor were located in 500 kb upstream and downstream of 3665 enhancer sites (Fig. S6D), demonstrating that HBV-miR-2 inhibitor was able to silence its corresponding enhancers and further downregulate oncogenic genes for HCC progression.

To further evaluate the specificity of HBV-miR-2 silencing effects, we compared the 1305 upregulated genes in HBV-infected HepG2-NTCP cells with the 650 downregulated genes by HBV-miR-2 inhibitors and found that there were 369 genes around 3665 enhancer sites with altered H3K27ac modification, which were specifically downregulated by HBV-miR-2 inhibitor from HBV infection through silencing enhancer activities. Importantly, these 369 genes were enriched in angiogenesis (*CD34*), cell cycle (*CCNA2*, *CCND1*), cell adhesion and migration, inflammatory response, collagen fiber and ECM organization (*COL1A1* and *LOXL2*) (Fig. 5C), which were crucial for the malignant progression of HCC [76–78]. Consistently, HBV-miR-2 inhibitor could clearly decrease the enrichment of H3K27ac at enhancer sites around the *COL1A1*, *CD34*, *LOXL2*, *CCNA2*, *TERT*, and *CCND1* genes in comparison with HBV infection (Fig. 5D and Fig. S6E), and further these downregulated genes validated by RT-qPCR (Fig. 5E). In conclusion, HBV-miR-2 functions as a dominant factor to drive HCC progression via upregulating gene expression dependent on host enhancer and HBV-miR-2 inhibitor could silence enhancer activities of HBV-SITE-1 to block HCC progression.

### Blocking HBV-SITE-1 represses oncogenic gene activation

**Fig. 6:**
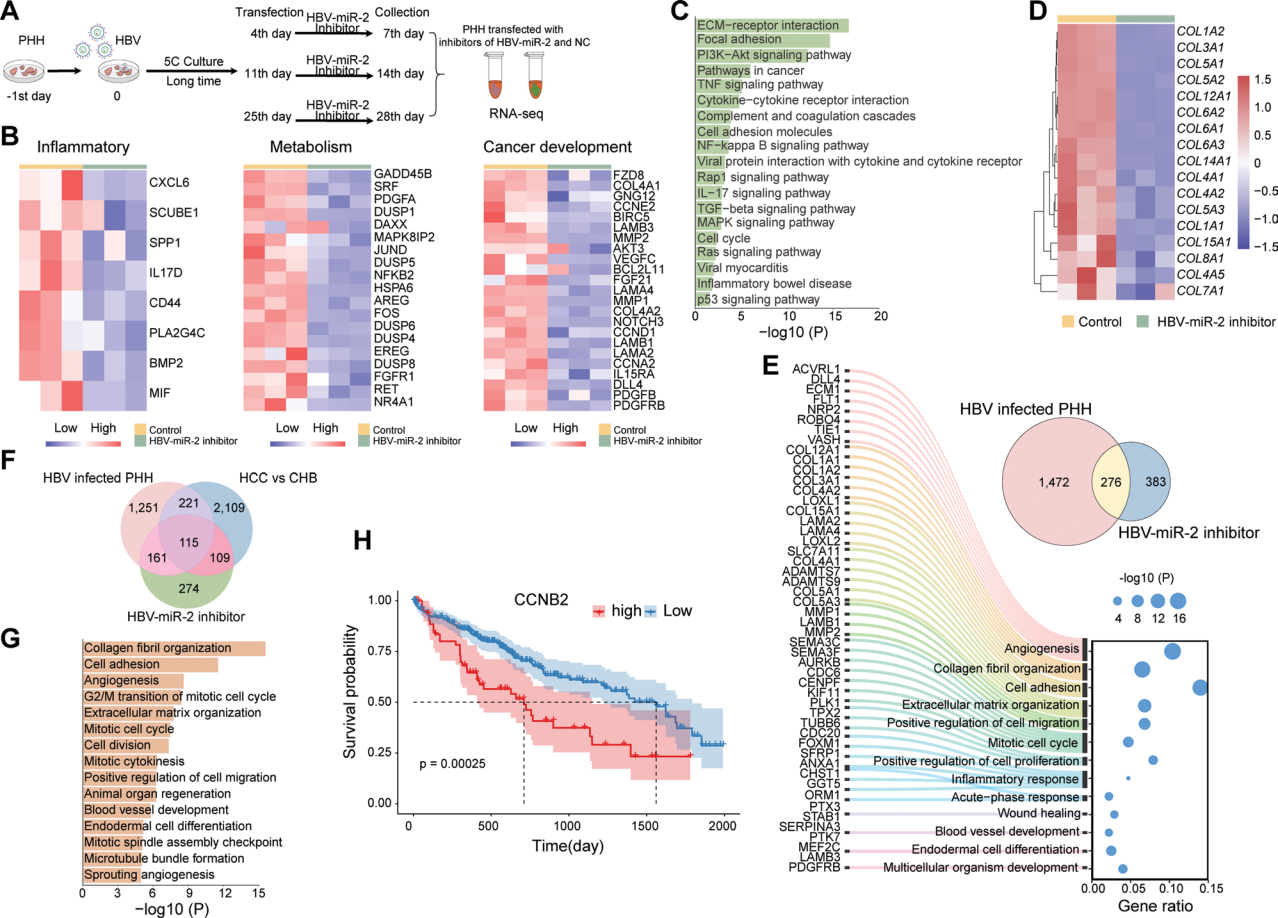
Targeting HBV-SITE-1 suppresses gene activation induced by HBV infection. **A** Schematic of the HBV-miR-2 inhibitor in HBV-infected PHH cells. HBV-miR-2 inhibitor transfected PHH cells at 4th, 11th and 25th, and these cells were collected to conducted RNA-seq at the 7th day, 14th day, and 28th day. **B** HBV-miR-2 inhibitor sequentially downregulated the inflammatory, metabolism, and cancer development-related genes in HBV infected PHH cells at the 7th day, 14th day, and 28th day. **C** KEGG analysis of 659 downregulated genes by HBV-miR-2 inhibitor in HBV infected PHH 28 days. **D** HBV-miR-2 inhibitor particularly decrease expression of genes encoding collagen. **E** Sankey diagrams displaying the biological function of the specific 276 genes. These 276 selected genes were upregulated by HBV infection and downregulated by HBV-miR-2 inhibitor treatment in HBV infected PHH cells 28 days. **F** 115 genes downregulated by HBV-miR-2 inhibitor were both upregulated in HBV infected PHH cells and HCC clinical samples. **G** GO enrichment analysis of these 115 genes. **H** Survival analysis curve of *CCNB2* in HCC

PHH are ideal to investigate the function of human liver in vitro, and we have established 28-day HBV-infected PHH models to mimic HCC progression and further demonstrated that the enhancer activities of HBV-SITE-1 could be largely silenced by HBV-miR-2 inhibitor. Therefore, we applied HBV-miR-2 inhibitor in this 28-day HBV-infected PHH models to investigate whether HCC development could be indeed blocked through silencing the integrated enhancer activities. Then, we transfected HBV-miR-2 inhibitors into HBV-infected PHH cells at the 4th, 11th, 25th day and collected cells at the 7th day, 14th day, and 28th day which represented the corresponding stages of HCC development (Fig. 6A). We found that HBV-miR-2 inhibitor could downregulate HBV-miR-2 itself as well as the confirmed upregulated genes (such as *CCNA2*, *MMP1*) by HBV infection (Fig. S7A), which all proved that our experiment system worked as expected. Specifically, inhibitor-HBV-miR-2 could down-regulate the expression of 127 genes on the 7th day, 568 genes on the 14th day, and 659 genes on the 28th day respectively induced by HBV infection. In detail, HBV-miR-2 inhibitor was able to downregulate inflammatory, metabolism, and cancer development-related genes at the 7th day, 14th day, and 28th day (Fig. 6B). Clearly, on the 7th day after HBV-miR-2 inhibitor treatment, these downregulated 127 genes were enriched in metabolic response, inflammatory response, cell adhesion and migration, angiogenesis, and cell proliferation (Fig. S7B). Surprisingly, among these 127 genes, there were 98 genes (77%) specifically induced by HBV-miR-2 in PHH (Fig. S7C), revealing that HBV-miR-2 and its inhibitor are specific-paired effector and silencer. Furthermore, GO analysis showed that HBV-miR-2 inhibitor, in the middle of HCC progression, was able to reduce genes related to HCC on the 14th day (Fig. S7D). Importantly, the 659 downregulated genes at 28 days presented closer connection with HCC by the finding that these genes were enriched in cell adhesion, collagen fiber assembly, angiogenesis, cell proliferation and migration (Fig. 6C and Fig. S7E). Specially, HBV-miR-2 inhibitor could dramatically suppress a set of genes encoding collagen proteins, which were well-known related to liver fibrosis and HCC (Fig. 6D). Furthermore, 276 genes induced by HBV infection at 28th days were specifically decreased by HBV-miR-2 inhibitor, which were associated with angiogenesis, collagen fibril and ECM organization, cell proliferation and migration, and inflammatory response (Fig. 6E), indicating that HBV-miR-2 inhibitor targeting HBV-SITE-1 could block HCC progression. Of note, the excessive accumulation of ECM proteins can induce liver fibrosis and accelerate the progression of HCC [79]. Meanwhile, HBV infection directly caused the significant upregulation of the collagen fibril genes (*COL12A1*, *COL1A1*, *COL3A1*, *COL1A2*, *COL5A1*, and *COL5A3*) and extracellular matrix organization genes (*COL15A1*, *COL4A2*, *COL4A1*, and *COL8A1*), and clearly, HBV-miR-2 inhibitor could block the upregulation of these genes (Fig. S7F), highlighting that HBV-SITE-1 resulting in the liver fibrosis by affecting ECM gene upregulation could be reversed by HBV-miR-2 inhibitor.

In addition to the HBV-infected PHH models, we expected that HBV-miR-2 inhibitor could also downregulate genes derived from clinical HCC GEO databases. Expectedly, among the 659 genes, 224 genes (34%) presented in HCC patients were significantly reduced by inhibitor-HBV-miR-2, further supporting that HBV-miR-2 inhibitor could suppress the transformation from chronic hepatitis to HCC. Additionally, among these 224 genes, 115 genes were also simultaneously up-regulated in PHH after HBV infection 28 days, which closely contributes to liver fibrosis and tumor development (Fig. 6F and G). Furthermore, 62 out of the 115 genes were expected to have high expression levels in TCGA HCC samples (Fig. S7G), including *COL1A1*, *LOXL2*, *CCNB2*, *STC2*, *KIF11*, *CD34*, *CDK1*, and *COL4A1*, which were reported as oncogenic genes to HCC development [80–82] and also associated with shorter survival (such as *CCNB2*, *SLC1A4*) (Fig. 6H and Fig. S7H).

Taken together, targeting HBV-SITE-1 was able to inhibit gene expression related to the progression of chronic hepatitis, liver cirrhosis, and HCC caused by HBV infection, supporting that silencing enhancer activities of HBV-SITE-1 by HBV-miR-2 inhibitor can be used for blockade of HBV-induced HCC progression.

### Targeting HBV-SITE-1 systemically in vivo prevents HCC pathological processes

**Fig. 7:**
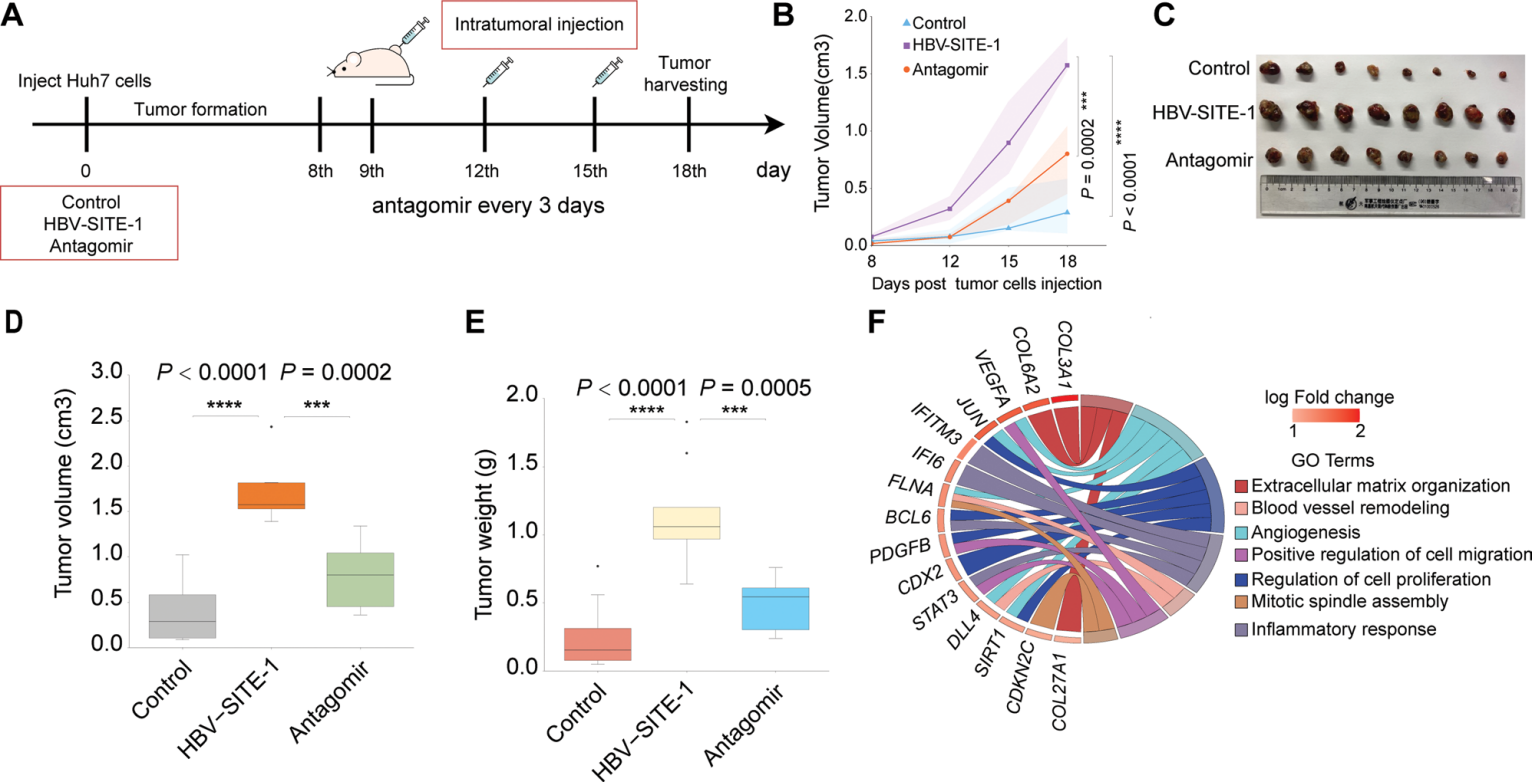
Intratumor treatment of HBV-miR-2 antagomirs inhibit tumor growth in vivo. **A** Schematic of the process of xenograft tumor model experiments. Intra-tumoral injection of HBV-miR-2 antagomir conducted at 9th, 12th and 15th day, and harvested at 18th day. **B** Growth curve by measuring the volume of tumors in mice by days. **C** Tumor tissues obtained at 18 days. The tumor sizes decreased by HBV-miR-2 antagomir. **D-E** Volume (D) and weight (E) of tumors decreased by HBV-miR-2 antagomir. **F** GO analysis of downregulated genes by HBV-miR-2 antagomir

As we have shown that HBV-miR-2 inhibitor could repress tumorigenic genes of HCC development in PHH cells through silencing enhancer activities, we wanted to investigate whether HBV-miR-2 antagomir could block HBV-induced disease progression in mouse models. Firstly, we constructed stable Huh7 cell lines by transfected HBV-SITE-1, and subsequently established the xenograft tumor models by injecting them into the flanks of BALB/c nude mice (Fig. 7A). Then, we performed intra-tumoral injection with HBV-miR-2 antagomirs every 3 days after tumor formation in mice. Clearly, HBV-SITE-1 could significantly accelerate the growth of tumors while antagomirs could restrain the tumor growth along with time (Fig. 7B). Meanwhile, the size, volume, and weight of tumor were significantly reduced by antagomir at the endpoint of 18 days (Fig. 7C-E), which might be owing that antagomirs could silence enhancer activities of HBV-SITE-1 represented by the low expression of HBV-miR-2 (Fig. S8A). Moreover, HBV-miR-2 antagomir could remarkably decrease the percentage of the Ki67 positive Huh7 cells (Fig. S8B and S8C), suggesting that HBV-miR-2 antagomir could restrain tumor growth by inhibiting cell proliferation. Previously, we have demonstrated that HBV-miR-2 transcribed from HBV-SITE-1 could upregulate angiogenesis-related genes in PHH cells through enhancers. Thus, we evaluated the angiogenesis in mice using CD34 marker [83]. As expected, we found that HBV-miR-2 antagomir could evidently inhibit the angiogenesis of tumor tissues (Fig. S8D). Notably, RNA-seq using these tumor tissues clearly demonstrated that HBV-miR-2 antagomir could downregulate genes in terms of angiogenesis, blood vessel remodeling, extracellular matrix organization, cell migration and proliferation (Fig. 7F). These findings suggested that targeting HBV-SITE-1 could disturb the tumor development through modulating cell proliferation and angiogenesis in vivo.

**Fig. 8:**
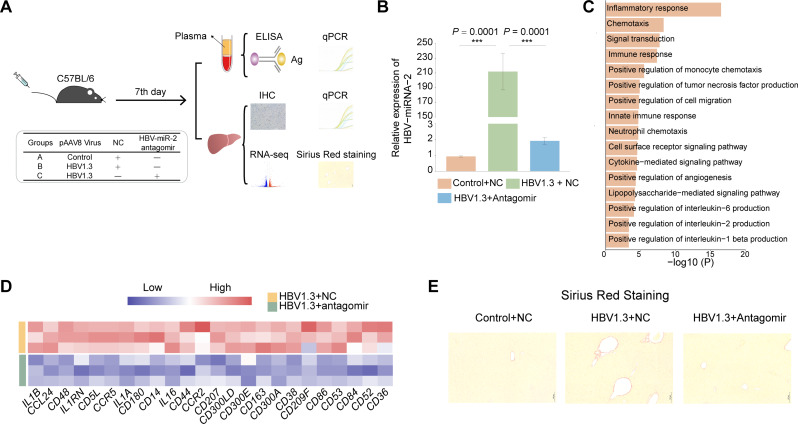
Intravenous treatment of HBV-miR-2 antagomirs prevent HBV-induced disease progression in vivo. **A** Schematic design in HDI models with systematically HBV-miR-2 antagomir. The liver and plasma samples were obtained to perform ELISA and RT-qPCR after 7 days. **B** HBV-miR-2 could be specifically downregulated by HBV-miR-2 antagomir in HDI models. **C** GO analysis of HBV-miR-2 antagomir downregulated genes related to inflammation in mouse liver. **D** Heatmap showing the inflammatory-related genes by HBV-miR-2 antagomir in HBV-infected mouse livers. **E** HBV-miR-2 antagomir reduces the accumulation of collagen fibers in mouse liver by Sirius red staining

To further explore whether intravenous injection of HBV-miR-2 antagomir could be applied in mice as systemic administration to block the HBV-induced HCC development in vivo, we established a HDI mouse model using the adenovirus pAAV8-HBV1.3 plasmid [84, 85] (Fig. 8A). The HBV DNA and HBV antigens in mouse plasma were detected in higher levels, and IHC assay showed that HBsAg antigens of HBV were presented in mouse liver tissues, indicating that the model was successfully constructed (Fig. S8E and S8F). Meanwhile, we found inflammatory genes including *IFIT1* and *IL6RA* were induced in mouse livers of HBV-infected mouse models (Fig. S8G). As is known, hepatic inflammation caused by HBV infection was the initial driver of HCC development, thus this HDI model was able to evaluate the effects of intravenous injection of HBV-miR-2 antagomirs on HBV-induced disease progression. Accordingly, HBV-miR-2 antagomirs could observably reduce the expression of HBV-miR-2 induced by HBV infection in HDI models (Fig. 8B). Surprisingly, we found HBV-miR-2 antagomir could specifically reduce the genes enriched in terms of inflammatory response, chemotaxis, as well as cell migration and angiogenesis, including *IL16*, *IL1A*, *CCL24*, *CD5L*, *CD86*, and *CD53* evaluated by RT-qPCR, highlighting that the systemically administration of HBV-miR-2 antagomir was able to block the inflammation induced by HBV infection (Fig. 8C-D and Fig. S8H). In addition, we also found HBV-miR-2 antagomir could reduce the accumulation of collagen fibers in mouse liver by Sirius red staining (Fig. 8E), demonstrating that targeting HBV-SITE-1 could cut down the collagen deposition caused by HBV infection. In summary, HBV-miR-2 antagomir could serve as an effective strategy to reduce inflammatory response and collagen fiber synthesis in response to HBV infection.

Taken together, our established two mouse models demonstrated that treatment of HBV-miR-2 antagomirs targeting HBV-SITE was able to efficiently inhibit HCC growth, and systemically administration of HBV-miR-2 antagomirs could be applied for clinical therapy to block the disease progression from chronic hepatitis to liver fibrosis and HCC.

## Discussion

HBV infection can lead to three stages of disease progression including chronic hepatitis, liver cirrhosis and hepatocellular carcinoma [86, 87]. HBV genomic integration is considered to provide additional growth advantage to support the clonal cell proliferation for HCC initiation by resulting in genomic instability and insertional mutagenesis [88]. Besides, viral proteins of HBV such as HBx and HBV core were another pathogenic factor for hepatocarcinogenesis via affecting cell functions and inflammatory responses [9, 89]. However, it remains uncertain that how HBV infection leads to the HCC progression. Here, we recognized the HBV fragments as HBV-SITEs integrated into human enhancer regions and acted as enhancers promoting on tumorigenic phenotypes by activating oncogenic genes. Surprisingly, only single nucleotide variation of HBV-SITE-1 almost abolished its effect, highlighting the significant tumorigenicity of HBV integrated fragment itself. Subsequently, we established a 28-day HBV-infected PHH models and found that specific gene expression patterns at three stages of HBV-infected PHH models could mimic the progression of HCC, which was further validated by GEO clinical transcriptome database covering CHB, liver cirrhosis and HCC. Thus, this long-term HBV-infected model provides an alternative approach to explore how HBV infection causes HCC progression and to further explore strategies to block HCC development. Finally, we demonstrated that targeting HBV-SITE-1 could significantly inhibit the HCC growth and inflammation caused by HBV infection through silencing the enhancer activities, providing a potential solution for HBV-induced diseases.

Currently, HBV oncogenicity mainly focuses on the genomic instability caused by HBV fragments integrated into human particular genome sites and the encoded proteins of HBV [6]. In this study, we revealed that transcripts of HBV-SITEs could facilitate HCC development through influencing the host transcriptome after HBV infection. Clearly, we demonstrated that integration events occurred in our HBV-infected PHH cell models, including the insertion points around the oncogenic genes such as *TERT*,* CCNA2* and *CCNE1* [6]. Further analysis on these integration events showed that HBV fragments preferentially integrated into human enhancer regions at different chromatins, termed as HBV-SITEs. In line with our findings, HBV DNA preferentially contacts with the host DNA at active chromatin regions marked by H3K27ac [90]. Interestingly, HBV-SITEs possessed enhancer activities by the findings that luciferase activities were intensive after different HBV-SITEs cloned into pGL3 plasmids, which may interact with the promoters of *TERT* and *CCNE1* to upregulate their expression in HBV-induced HCC patients [7]. Notably, NamiRNAs (such as miR-339, miR-26A1, and miR-492) transcribed from enhancer regions could modulate the tumor progression via regulating cell proliferation or migration [25, 91, 92]. Correspondingly, the transcripts of five HBV-SITEs were detected in four established HBV cell models. Furthermore, over 1500 genes activated by HBV-SITE-1 transcripts were involved in cell cycle and cell migration associated with tumor development, which further resulted in the increased proliferation and migratory abilities of HCC. Instead, blocking HBV-SITE-1 with its antisense nucleotides could significantly inhibit the cell proliferation and migration of HCC cells. Therefore, our findings indicated that the sequences of HBV-SITEs could serve as novel vital pathogenic elements for HCC development, which may expand our sights on the understanding of viral tumorigenicity.

Furthermore, epigenetic alterations could modulate gene expression pattern to trigger HCC [93, 94]. As the key regulatory epigenetic elements, enhancers could drive the continuous transcription of oncogenes during liver carcinogenesis [95]. In particular, HBx protein could affect H3K27ac enrichment to promote HCC progression via upregulating ETV4 expression [96]. Except this regulatory role of HBV proteins on enhancers, we found that both HBV-SITE-1 transfected HepG2 and Huh7 exhibited globally the significant enrichment of H3K27ac. Surprisingly, seed sequence of HBV-miR-2 embedded within HBV-SITE-1 was dramatically enriched in the motifs of these H3K27ac peaks, hitting the potential interaction between HBV-SITE-1 and host enhancers. Specially, HBV-SITE-1 remarkably contributed to the H3K27ac enrichment at the candidate enhancer around *CDK8*, whose enhancer activities were verified by dual-luciferase reporter assay. Meanwhile, co-transfection of HBV-SITE-1 could further strengthen the luciferase activities mediated by *CDK8* enhancer. Moreover, knockout of the binding sites of HBV-miR-2 in human genome could restrain the activation of *CDK8* induced by HBV-SITE-1, demonstrating that interaction between HBV-SITE-1 and host enhancer could specifically activate oncogenic gene expression. In accordance with our results, miR-492 could stimulate epithelial-mesenchymal transition of pancreatic cancer via interacting with enhancer [92]. Consequently, as no-coding sequences, HBV-SITEs may be as enhancer trigger to alter the chromatin status and further promote HCC progression, which supported noncoding sequences of viruses have diverse oncogenic roles [10–12].

Moreover, our work greatly extends the understanding of pathogenic elements to determine the pathogenicity of diverse HBV genotypes. It is known that HBV has different genotypes with geographical distinctiveness and genotype H is associated to a less severe progression of disease [3]. Notably, less than 8% nucleotide sequence differences among these HBV genotypes [4]. Accordingly, we should pay more attention on these minor nucleotide variations, which may largely affect their viral pathogenicity. As expected, we found the single nucleotide variation (T> G) of HBV-SITE-1 in the H genotype almost abolished its pathogenicity on cell proliferation and migration for HCC. To our surprise, this nucleotide variation coincidently appeared at the seed sequences of HBV-miR-2 embedded in HBV-SITE-1, which may destroy their interaction with host enhancer. As a result, HBV-SITE-1 of H genotype didn’t activate the oncogenic genes for tumorigenesis. These findings demonstrated that nucleotide variations at HBV-SITEs could determine the virulence of HBV genotypes, suggesting that HBV-SITEs could serve as alternative therapeutic targets for HBV-induced HCC. Importantly, the discovery of HBV-SITEs may provide a novel insight for exploring the other oncogenic viruses with host genomic integration.

Besides, HBV DNA integration induced genome instability has long been considered to be the predominant contributor to liver tumorigenesis [97, 98]. Particularly, these HBV integrations could result in a substantial enhancement of distinct oncogenic genes, which may have a profound influence on phenotypic characteristics [39]. Current antiviral treatments exist the problem such as the large adverse reactions, viral resistance, and inability to clear cccDNA leading to HBV rebound [15, 99], eventually cannot block function of integrated HBV sequences [100, 101]. We have found HBV-SITE-1 could act as an enhancer to upregulate oncogenic genes caused by HBV infection and presented higher levels in HCC, highlighting that HBV-SITE-1 embedded with HBV-miR-2 largely serves as the vital pathogenic factor of HCC progression. Therefore, we designed an inhibitor targeting HBV-SITE-1 and found this inhibitor could decrease the enhancer activity of HBV-SITE-1 and downregulate oncogenic genes involved in the HCC progression. In this case, silencing the enhancer activity of HBV-SITE-1 would be a promising strategy for blocking HCC progression.

Nowadays, how to prevent the development of HBV-induced HCC is still a clinical challenge [102, 103]. Our results revealed that targeting HBV-integrated sequences can be the preferable choice to block HCC development. Meanwhile, antagomirs via intravenous injection or intratumor injection are practicable therapeutic approaches for HCC. We designed animal experiments and demonstrated that antagomir targeting HBV-miR-2 embedded in HBV-SITE-1 was able to block HBV-induced disease progression in vivo. Primarily, HBV-miR-2 antagomir could inhibit the pro-tumor gene activation and reduce the HCC growth via inhibiting cell proliferation in xenograft tumor models. Secondly, the systematically intravenous injection of HBV-miR-2 antagomir could improve the inflammation and collagen deposition of liver caused by HBV, highlighting that silencing HBV-miR-2 is a prospective therapeutic strategy for reducing the pathogenicity of HBV integration. This antagomir-based intravenous injection was an effective administration for small nucleotide drugs to fight against HBV-related diseases.

In addition, there are still some limitations in our research. Honestly, it is difficult for us to directly evaluate the therapeutic potential of antagomirs targeting HBV-SITEs due to the lack of the animal models of HBV-induced HCC, which should be verified after the models are successful establishment. In this case, we may try to construct a long-term HBV infected mouse model with pAAV-HBV or pAAV- HBV-SITEs to induce HCC and treat them with antagomirs after models constructed in the future, which will provide more powerful evidence to confirm the oncogenic roles of HBV-SITEs. Meanwhile, it deserves us effort to explore the exact mechanism how HBV-SITEs binds to host enhancer to activate the oncogenic genes for HCC development. Importantly, we have expanded this oncogenic mechanism to other cancer caused by DNA virus such as HPV induced cervical cancer, which may open a new perspective for the study of virus-related tumors.

## Conclusion

HBV-SITEs could epigenetically activate host oncogenic gene transcription and facilitate hepatocellular carcinoma development as enhancer triggers, which provides promising therapeutic targets for HBV-induced HCC and may present an extraordinary oncogenic model for other oncogenic DNA viruses. And design of nucleotide drugs against HBV integrated fragments such as HBV-SITEs could be potential therapeutic solution to clinically prevent their oncogenic function through blocking their biological function as enhancer triggers, which highlights a novel potential strategy for the treatment of DNA viruses-induced cancers.

## Electronic supplementary material

Below is the link to the electronic supplementary material.

Supplementary Material 1

## Data Availability

The datasets used and analyzed during the current study are available from the corresponding author on reasonable request.
