# Supplementary material for: HBV sequence integrated to enhancer acting as oncogenic driver epigenetically promotes hepatocellular carcinoma development
Source: J Exp Clin Cancer Res. 2025 May 22;44:155. doi: 10.1186/s13046-025-03413-8 (PMC12096768; doi:10.1186/s13046-025-03413-8)
Supplement: Supplementary file 1 — Supplementary Material 1 [file 13046_2025_3413_MOESM1_ESM.docx]

**Supplementary Material 1.**


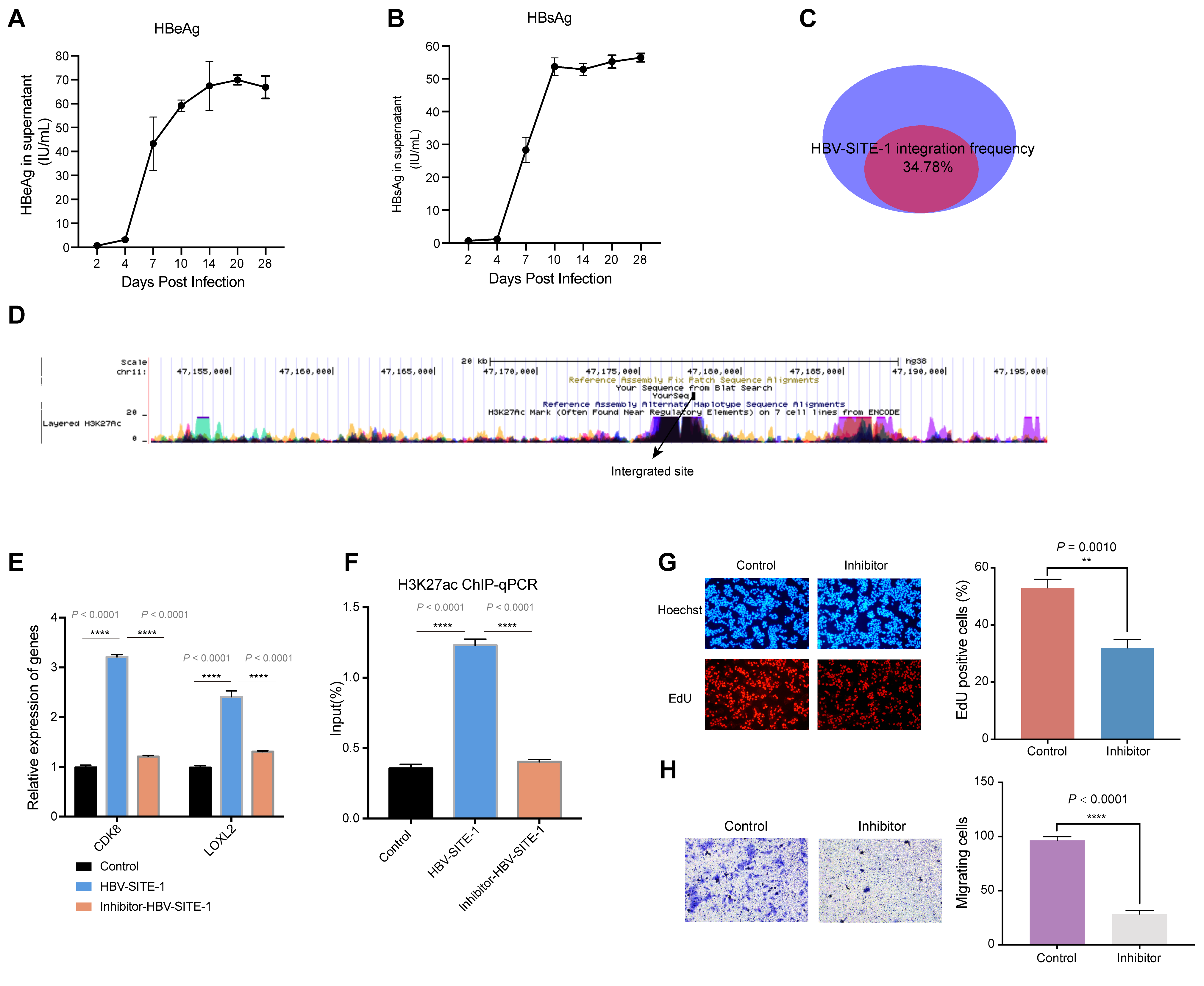


**Fig.S1** Targeting HBV-SITEs inhibit cell proliferation and migration by decreasing enhancer activity. **A** HBeAg antigens detected by ELISA at different times of HBV infected PHH cells. **B** HBsAg antigens detected by ELISA at different times of HBV infected PHH cells. **C** The integration frequency of HBV-SITE-1 in HBV-infected HepG2-NTCP cells. **D** The integrated HBV-SITE-1 was also prone to insert into human enhancer regions in HBV-infected HepG2-NTCP cells. **E-F** The gene expression (E) and enhancer activity (F) detected after inhibiting HBV-SITE-1 by RT-qPCR (E) and ChIP-qPCR (F). **G-H** Changed proliferation (G) and migration ability (H) of liver cancer cells after inhibition of HBV-SITE-1.


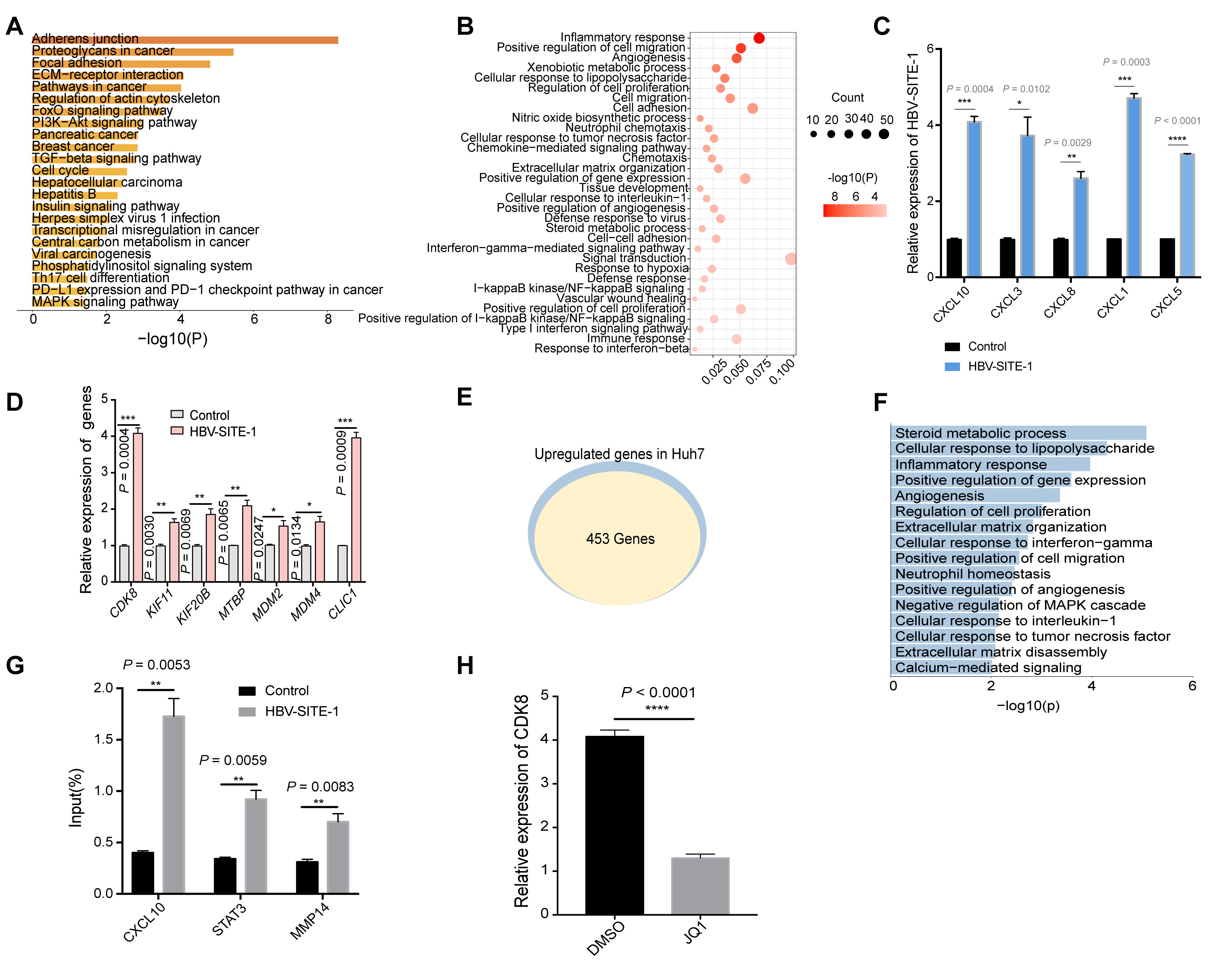


**Fig.S2** HBV-SITEs upregulate genes related to HCC through changing enhancer activity.

**A** KEGG Pathway Enrichment analysis of upregulated genes in HepG2 cells transfected HBV-SITE-1. **B** GO enrichment analysis of upregulated genes in Huh7 cells overexpressed transfected HBV-SITE-1. **C** The expression of inflammation-related genes detected after HBV-SITE-1 transfected in Huh7 cells by RT-qPCR. **D** The expression of cell cycle genes after HBV-SITE-1 transfected by RT-qPCR. **E** 453 upregulated genes were potentially regulated by HBV-SITE-1 through enhancers in Huh7 cells. **F** GO analysis of those 453 upregulated genes by DAVID. **G** The enhancer activity of these upregulated genes detected in Huh7 cells transfected HBV-SITE-1 by ChIP-qPCR. **H** *CDK8* gene expression after JQ1 treatment by RT-qPCR results.


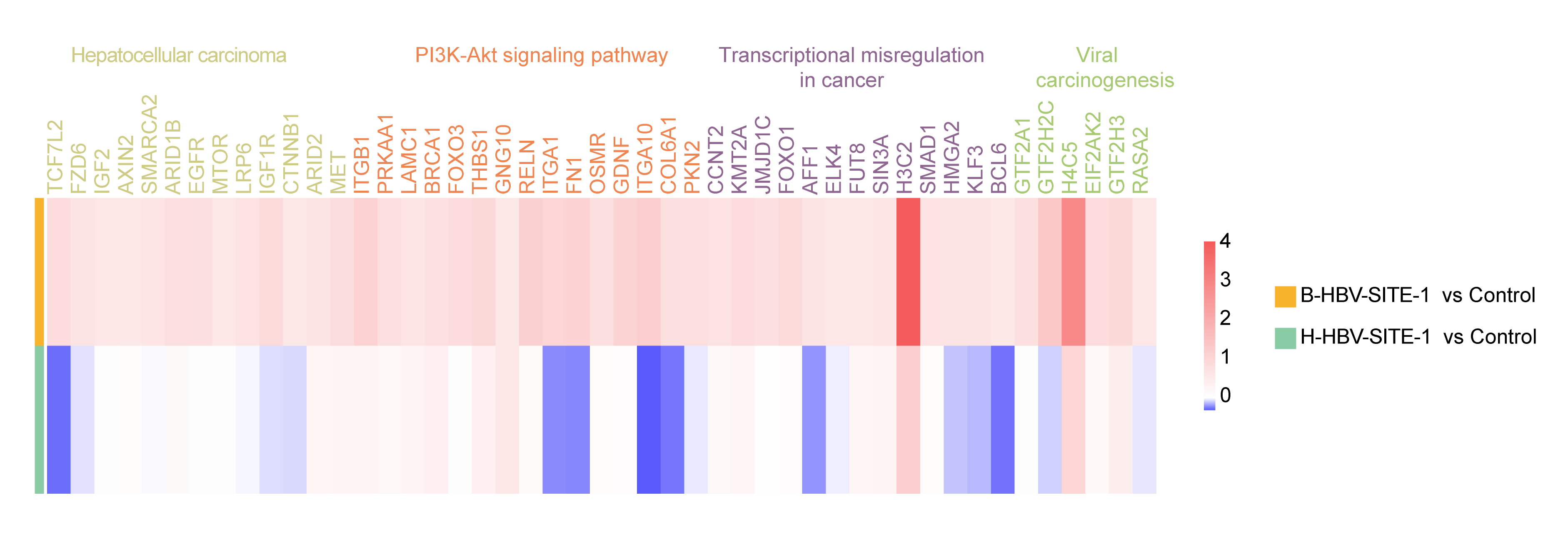


**Fig.S3** H-HBV-SITE-1 could not upregulate genes related to HCC compared with B-HBV-SITE-1 transfected. Among the 113 genes, 47 genes displayed were not upregulated by H-HBV-SITE-1 compared to B-HBV-SITE-1.


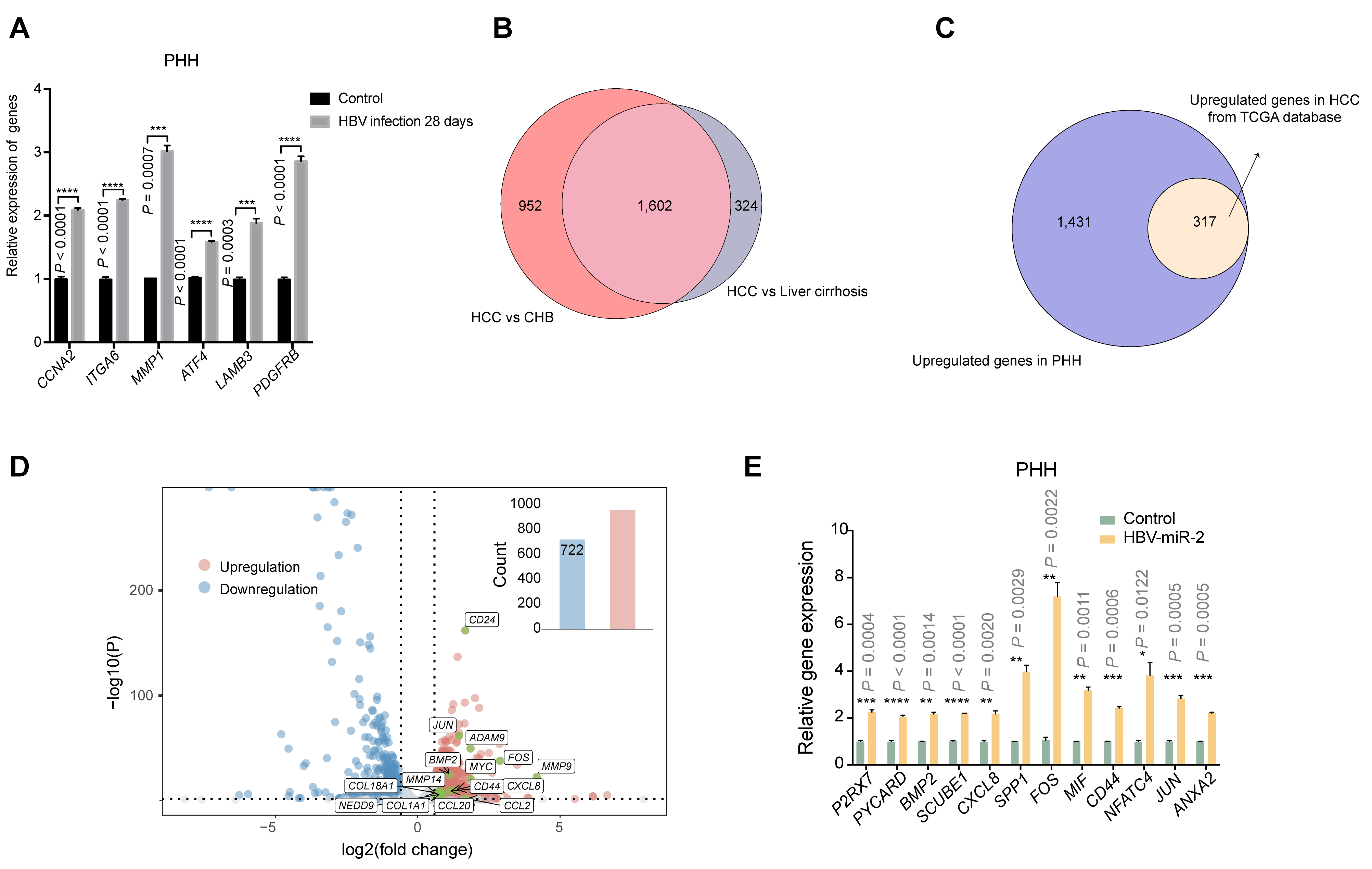


**Fig.S4** HBV-infected PHH 28 days upregulated many genes related to HCC development and progression. **A** Genes related to HCC were up-regulated on the 28th day of HBV infection of PHH by RT-qPCR. **B** There were 2554 genes upregulated in HCC compared with chronic hepatitis and 1926 genes upregulated in HCC compared with liver cirrhosis. 1602 genes were overlapped between 2554 and 1926 genes. **C** 317 genes upregulated in HBV infection PHH 28 days also presented higher expression levels in HBV-related HCC of TCGA database. **D** Volcano plot on the transcriptome in PHH cells transfected with HBV-miR-2. The histogram shows 957 upregulated genes induced by HBV-miR-2 transfection in PHH. The boxes exhibit the upregulated oncogenic genes marked in green were related to HCC development. **E** Inflammatory and fibrosis genes validated by RT-qPCR in PHH transfected with HBV-miR-2.


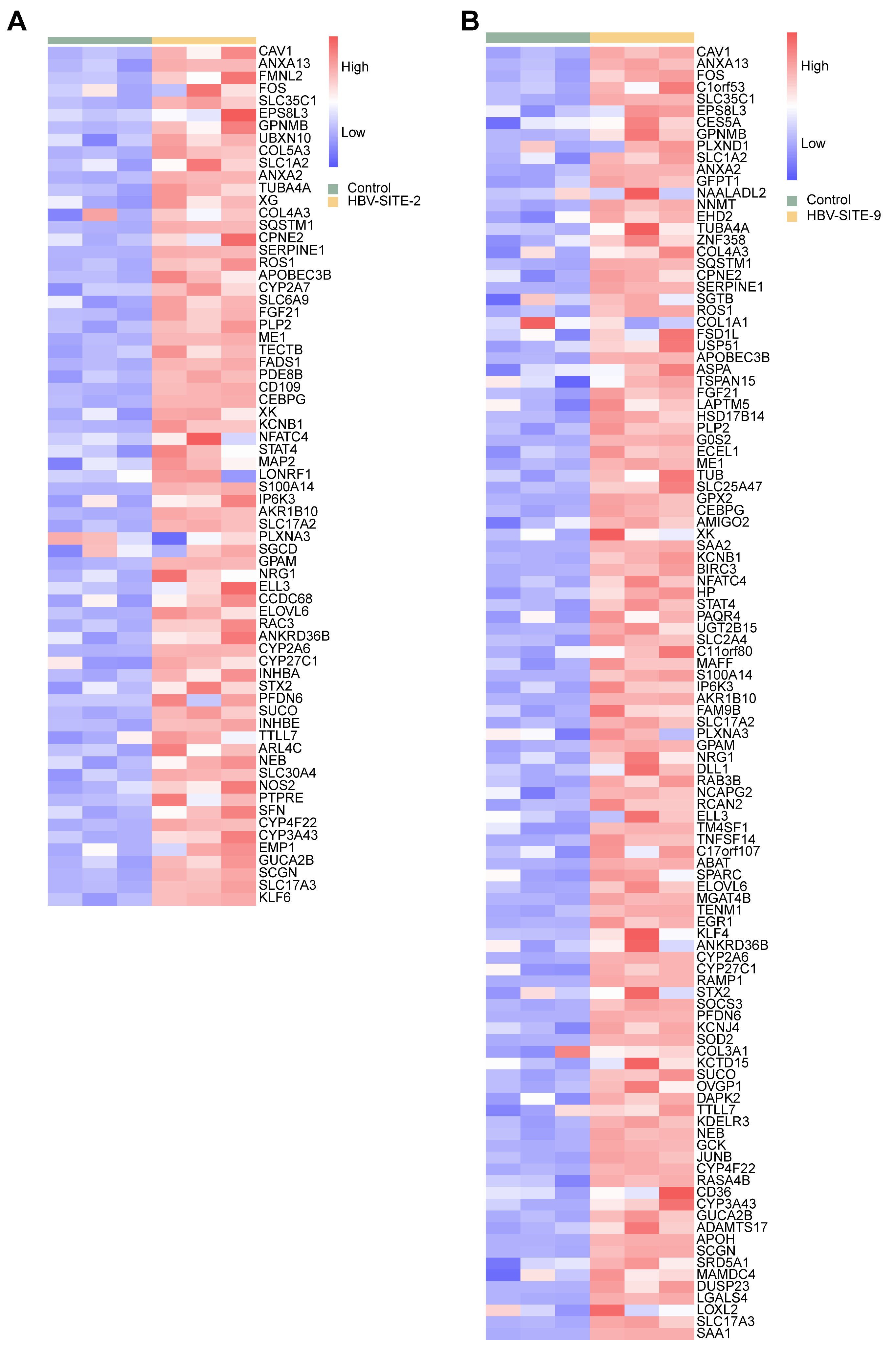


**Fig.S5** HBV-SITEs contribute to HBV pathogenic by upregulated genes overlapped with HBV infection. **A** HBV-SITE-2 could upregulate 69 genes overlapped with HBV infection in PHH cells. **B** HBV-SITE-9 could upregulate 110 genes overlapped with HBV infection in PHH cells.


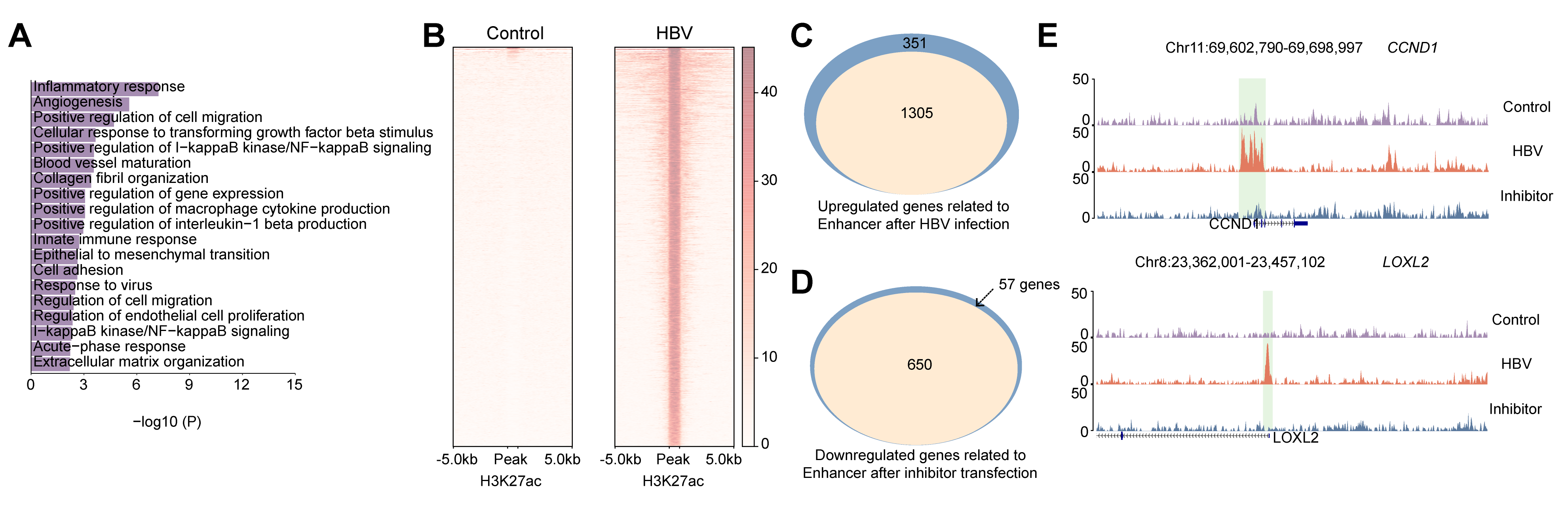


**Fig.S6** HBV-miR-2 upregulated genes related to HCC through targeting host enhancers. **A** Enrichment analysis of the 368 genes both upregulated by HBV infection in HepG2-NTCP cells and PHH cells. **B** Profiling of H3K27ac enrichments in HBV infected HepG2-NTCP cells. Each row represents one peak centered at the midpoint between two 5kb flanking sequences. **A total** of 4362 H3K27ac peaks are shown in HBV-infected HepG2-NTCP with transfection of the control (left) or HBV infection (right). **C** Among the 1656 upregulated genes, 1305 genes were upregulated through enhancer H3K27ac change during HBV infection in HepG2-NTCP cells. **D** Venn showing the downregulated genes with lower enhancer activities in HBV-infected HepG2-NTCP cells. 650 downregulated genes in HBV-infected HepG2-NTCP cells with HBV-miR-2 inhibitor transfection, are surrounding the enhancer regions with decreased H3K27ac enrichment. **E** IGV visualization of H3K27ac peaks in HBV-infected HepG2-NTCP cells. The regions on the alteration of enhancer activities are marked in light green boxes. HBV-miR-2 could reverse the enhancer activity of *CCND1* and *LOXL2* induced by HBV infection.


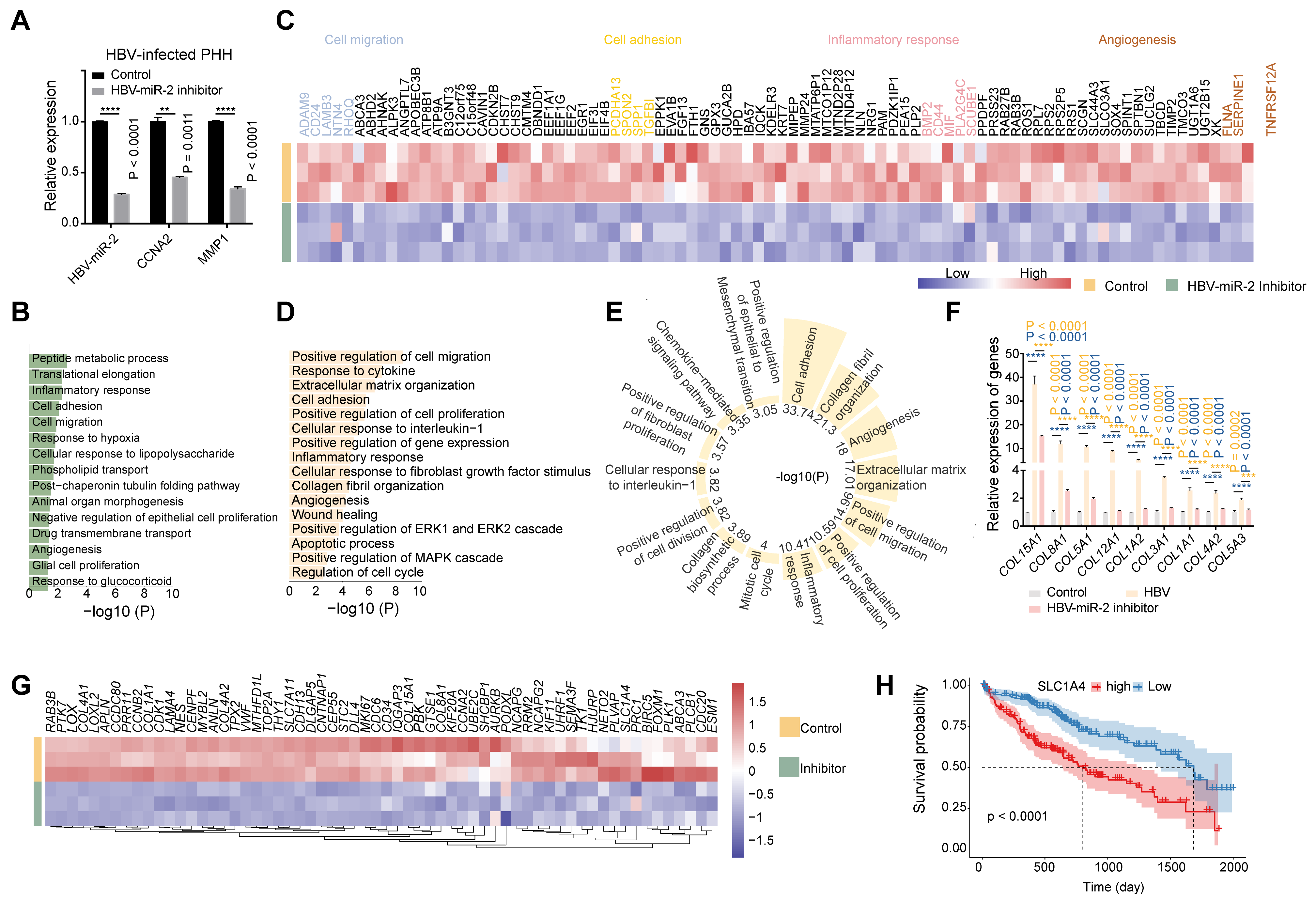


**Fig.S7** Inhibitor of HBV-miR-2 downregulated genes related to HCC in HBV-infected PHH cells and HepG2-NTCP cells. **A** HBV-miR-2 inhibitor could downregulate the expression of HBV-miR-2 and genes caused by HBV infection by RT-qPCR. **B** GO enrichment analysis showed the functions of 127 genes downregulated by inhibitor-HBV-miR-2 in HBV-infected PHH 7 days. **C** HBV-miR-2 inhibitor downregulated 98 genes induced by HBV-miR-2 in HBV-infected PHH 7 days. **D** The Enrichment analysis of 568 genes downregulated by HBV-miR-2 inhibitor on the 14th day after HBV infection. **E** GO analysis of 659 downregulated genes with HBV-miR-2 inhibitor treatment in HBV-infected PHH 28 days. **F** Collagen fibril genes and extracellular matrix organization genes can be downregulated by HBV-miR-2 inhibitor detected by RT-qPCR. **G** 62 genes downregulated by HBV-miR-2 inhibitor also exhibited a higher level in TCGA HCC samples and these genes were displayed in heatmap. **H** The survival analysis curve showed that high expression of *SLC1A4* was associated with shortened survival in HCC.


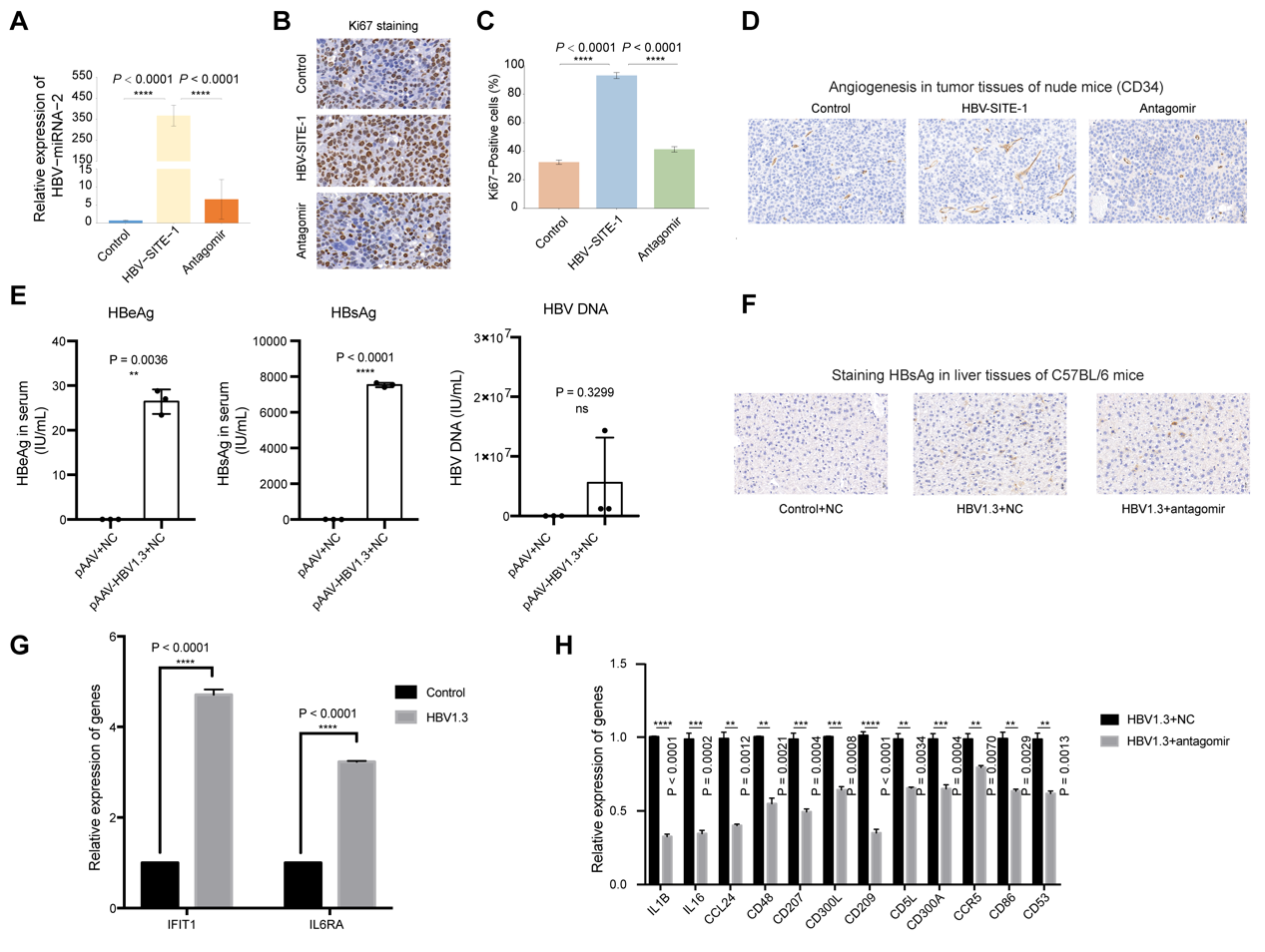


**Fig.S8** Antagomir of HBV-miR-2 decreased tumor growth and inflammation of the liver *in vivo*.

**A** HBV-miR-2 antagomir could decrease the expression of HBV-miR-2 in xenograft tissues by RT-qPCR. **B-C** HBV-miR-2 antagomir could decrease the percentage of the Ki67 positive cells by microscope (B) and by image J software calculation (C) within tumor tissue. **D** HBV-miR-2 antagomir could reduce the angiogenesis of tumor tissue induced by HBV-SITE-1 in mice by CD34 IHC staining. **E** The HBV DNA and the antigens in the plasma of mice were detected by qPCR and ELISA. **F** The HBsAg of mouse liver tissue detected by IHC experiments and the brown cells were HBsAg positive. **G** HBV infection induced inflammatory-related genes upregulated by RT-qPCR. **H** HBV-miR-2 antagomir could downregulate the expression of inflammatory-related genes by RT-qPCR in mouse liver.
